# Characterization and 3-Year Survival of Clinical Subtypes and Host Response Phenotypes of Acute Respiratory Failure Requiring Mechanical Ventilation

**DOI:** 10.1016/j.chstcc.2026.100274

**Published:** 2026-05-07

**Authors:** Georgios D. Kitsios, William G. Bain, John W. Evankovich, Niall Prendergast, Matthew Hensley, Tomeka Suber, Kyle Inman, Caitlin Schaefer, Melissa Saul, Xiaoping Chen, Menaal Haque, Janet Lee, Alicia Rizzo, Shulin Qin, Yingze Zhang, Alison Morris, Charles Dela Cruz, Seyed Mehdi Nouraie, Bryan J. McVerry, Faraaz A. Shah

**Affiliations:** Division of Pulmonary, Allergy, Critical Care and Sleep Medicine (G. D. K., W. G. B., J. W. E., N. P., M. H., T. S., K. I., C. S., M. S., X. C., A. R., S. Q., Y. Z., C. D. C., S. M. N., B. J. M., and F. A. S.), Department of Medicine, the Acute Lung Injury and Infection Center of Excellence (G. D. K., W. G. B., J. W. E., N. P., M. H., T. S., K. I., A. R., C. D. C., S. M. N., B. J. M., and F. A. S.), University of Pittsburgh; the Veterans Affairs Pittsburgh Healthcare System (W. G. C., B. J. M., and F. A. S.), Pittsburgh, PA; the George Washington University (M. H.), Washington, DC; the Washington University School of Medicine (J. L.), St. Louis, MO; and the Rutgers Robert Wood Johnson Medical School (A. M.), New Brunswick, NJ. B. J. M. and F. A. S. contributed equally to this manuscript.

**Keywords:** acute respiratory failure, ARDS, precision medicine, prediction, subphenotype

## Abstract

**BACKGROUND:**

Acute respiratory failure (ARF) requiring invasive mechanical ventilation affects approximately 1.5 million adults in the United States annually. Whereas ARDS is well characterized, non-ARDS ARF—comprising most patients who are mechanically ventilated—lacks systematic characterization of clinical features, biological profiles, and long-term outcomes.

**RESEARCH QUESTION:**

How do ARF subtypes differ in clinical, physiologic, and biological characteristics and in long-term survival?

**STUDY DESIGN AND METHODS:**

Prospective cohort study of 826 mechanically ventilated adults enrolled at a single academic center from 2011 through 2024. Patients were classified systematically into 7 predefined ARF subtypes using consensus methodology. We compared physiologic features, biological subphenotypes, and 3-year survival.

**RESULTS:**

Patients were classified as having ARDS (36.1%), being at risk for ARDS (ARFA; 34.1%), having airway control (10.7%), having other or multifactorial features (7.4%), having congestive heart failure (CHF; 6.2%), having acute-on-chronic hypercapnic respiratory failure (AoCHRF; 3.3%), and having acute exacerbation of interstitial lung disease (AEILD; 2.3%). Subtypes differed significantly in gas exchange (hypoxemia and ventilatory ratio), respiratory mechanics (normalized elastance), and radiographic severity (*P* < .01 for all). Plasma inflammatory biomarkers were highest in patients with ARDS and ARFA, with hyperinflammatory subphenotype prevalence ranging from 0% (AoCHRF) to 30.1% (ARFA). Three-year survival ranged from 10.5% (AEILD) to 67.0% (airway control): 37.7% (other or multifactorial), 48.1% (AoCHRF), 56.9% (CHF), 53.2% (ARFA), and 51.3% (ARDS). Hyperinflammatory subphenotype classification predicted lower survival (42.9% vs 54.9%; *P* < .01), predominantly in patients with ARDS and ARFA, whereas patients with other subtypes showed minimal subphenotype-associated survival differences.

**INTERPRETATION:**

Our results show that patients who are mechanically ventilated with ARF demonstrate marked heterogeneity in physiologic features, biology, and long-term outcomes. Overall, fewer than one-half of patients survived to 3 years, but survival varied markedly across clinical subtypes beyond traditional ARDS categorization. Clinical subtype classification identifies distinct populations with divergent long-term prognoses, providing information complementary to biological subphenotyping. These findings provide quantitative outcome data to support prognostic counseling and to establish a reproducible framework for clinical research in ARF resulting from heterogeneous causes.

Acute respiratory failure (ARF) requiring invasive mechanical ventilation (IMV) affects > 1.5 million adults in the United States annually, with incidence rising globally because of aging populations and increasing chronic disease burden.^1,2^ Despite advances in critical care, patients who are mechanically ventilated experience short-term mortality rates of 18% to 50% and substantial long-term morbidity among survivors.^3–5^ Yet, our understanding of clinical characteristics, biological profiles, and long-term outcomes in this population remains limited, particularly for most patients with ARF that does not meet criteria for ARDS.^3,5^

Research on patients who are mechanically ventilated has focused predominantly on ARDS, which has well-defined diagnostic criteria, evidence-based treatment guidelines, and extensive characterization of outcomes.^6,7^ However, ARDS represents only 10% to 30% of patients who are mechanically ventilated.^3,5^ Far less systematic investigation has been undertaken of the remaining majority of patients with non-ARDS ARF resulting from causes such as cardiogenic pulmonary edema, chronic lung disease exacerbations, airway protection needs, and interstitial lung disease.^8^ Non-ARDS ARF typically is defined by the absence of ARDS, rather than the presence of distinct clinical entities, resulting in critical knowledge gaps. Clinicians lack subtype-specific data on biological profiles, clinical trajectories, and particularly long-term outcomes to guide prognostication, to inform treatment intensity decisions, and to design targeted clinical trials. Moreover, enrolling heterogeneous ARF populations with divergent pathophysiologic features risks diluting treatment effects and obscuring mechanistic insights while the relationship between clinical subtypes and emerging biological subphenotypes (eg, hyperinflammatory vs hypoinflammatory profiles in ARDS)^9^ remains poorly defined across the broader ARF spectrum. These gaps limit precision medicine approaches in critical care and underscore the need for systematic classification of ARF beyond ARDS.

To address these gaps, we systematically classified mechanically ventilated adults with ARF into predefined clinical subtypes and sought: (1) to compare physiologic and biological characteristics across subtypes, including distribution of hyperinflammatory and hypoinflammatory subphenotypes; and (2) to determine survival through 3 years. We hypothesized that clinical subtypes defined through systematic clinical classification would differ in clinical and biological profiles and would demonstrate divergent long-term outcomes.

## Study Design and Methods

### Study Design and Population

We conducted a prospective cohort study of adults with ARF receiving IMV who were enrolled in a clinical registry and biospecimen repository at University of Pittsburgh Medical Center Presbyterian, Shadyside, and East hospitals from October 2011 through April 2024.^10–13^ The study was approved by the University of Pittsburgh institutional review board (Identifier: STUDY19050099), and informed consent was obtained from patients or their legally authorized representatives. All procedures followed the ethical standards of the Declaration of Helsinki. Eligible patients for this cohort were adults (18-90 years of age) admitted to medical or cardiac ICUs requiring IMV via endotracheal intubation for ARF resulting from diverse causes including pneumonia, sepsis, aspiration, cardiogenic pulmonary edema, chronic lung disease exacerbations, and airway protection needs. We excluded patients with chronic ventilator dependence via tracheostomy. Complete inclusion and exclusion criteria are provided in e-Table 1.

### ARF Subtype Classification

We systematically classified participants into 1 of 7 predefined ARF subtypes selected for their clinical relevance and construct validity in representing the spectrum of ARF presentations (Table 1): (1) ARDS according to Berlin definition criteria^14^; (2) ARFA, defined as having identifiable risk factor(s) for acute lung injury (e-Table 2), but not fulfilling ARDS diagnostic criteria^10,15^; (3) CHF, defined as having hypoxemic ARF resulting from cardiogenic pulmonary edema; (4) airway control, defined as being intubated for airway protection without primary pulmonary pathologic features; (5) AoCHRF, defined as having acute decompensation of chronic ventilatory failure resulting from obstructive airways disease; (6) AEILD, defined as having acute worsening of interstitial lung disease with diffuse radiographic abnormalities^16^; and (7) other or multifactorial, defined as patients not clearly fitting other categories or with overlapping features from multiple subtypes.

Classification was performed using a consensus methodology by a committee of board-certified critical care physicians who reviewed clinical data, chest imaging, and laboratory results while masked to research biomarkers and clinical outcomes. Participants underwent consensus classification at up to 3 time points during the first 10 days of study enrollment corresponding to protocol-based study visits for biospecimen sampling: baseline (day 1), middle (days 3-6), and late (days 7-10). For participants with multiple assessments, subtype classifications were assigned hierarchically to capture the most severe presentation: participants meeting ARDS criteria at any time point were classified as having ARDS, whereas ARFA classification required no progression to ARDS throughout the observation period. Complete details on longitudinal classification are provided in e-Appendix 1. From October 2011 through March 2020, consensus was achieved through in-person meetings with open discussion and majority vote. From October 2020 through April 2024, a standardized virtual consensus process was implemented using structured case presentations and anonymous online voting (e-Fig 1). Interrater reliability was assessed quantitatively for the virtual consensus cohort. Detailed consensus methodology, including the standardized case presentation format, questionnaires, and interrater reliability analyses, is provided in e-Tables 2 and 3 and e-Figures 2 and 3.

### Data Collection

We collected demographic data, comorbidities, vital signs, laboratory values, mechanical ventilation parameters at enrollment, as well as applied treatments during IMV course. Chest radiographs and CT scans (when available) were reviewed as part of the consensus classification process. From available baseline chest radiographs obtained on intubation, we quantified radiographic severity with the Radiographic Assessment of Lung Edema score, as previously described.^17^ We calculated modified Sequential Organ Failure Assessment (excluding neurologic component) scores at baseline.

### Biomarker Measurements and Biological Subphenotyping

Plasma samples collected at enrollment within 72 hours of intubation. Median time from intubation to sample collection ranged from 1.0 to 2.0 days across ARF subtypes. We measured host-response biomarkers using a customized 10-plex Luminex assay (R&D Systems) for markers of inflammation (eg, IL-6, IL-8, and soluble tumor necrosis factor receptor 1), endothelial injury (angiopoietin-2 [Ang-2]), epithelial injury (soluble receptor for advanced glycation end-products), and infection (procalcitonin).^10^ We classified patients into hyperin-flammatory vs hypoinflammatory subphenotypes using a previously validated parsimonious 4-biomarker logistic regression model developed by our group.^11^ This model, which was derived originally and validated internally in a subset of 498 participants from this cohort, incorporates baseline plasma procalcitonin, Ang-2, soluble tumor necrosis factor receptor 1, and serum bicarbonate. The model was applied without modification using the published coefficients and a predicted probability threshold of 0.5 to dichotomize subphenotypes.

### Outcomes

The primary outcome was survival through 3 years from enrollment, ascertained from the electronic medical record and the UPMC Electronic Centralized Data Repository. Secondary outcomes included duration of IMV and discharge destination for survivors.

### Statistical Analysis

We summarized patient characteristics by ARF subtype using medians with interquartile ranges for continuous variables and frequencies with percentages for categorical variables. Between-subtype comparisons used the Kruskal-Wallis test for continuous variables and the Fisher exact test for categorical variables. Comparisons of physiologic and biomarker variables excluded patients receiving extracorporeal membrane oxygenation because of the impact of the extracorporeal membrane oxygenation circuit and associated ventilatory settings on such parameters.^18^ Missing biomarker values were not imputed because of concerns about reliable prediction from available clinical covariates. Missing clinical variables were imputed using clinically informed methods leveraging physiologic relationships (eg, estimating Paco_2_ from venous blood gas values) and statistical imputations, when appropriate. Complete details are provided in e-Appendix 1. Survival was analyzed using Kaplan-Meier methods with log-rank tests to compare curves across subtypes. To assess whether ARF subtype classifications and host-response subphenotypes provided independent prognostic information beyond baseline patient characteristics, we performed multivariable Cox proportional hazards regression for the primary outcome of survival through 3 years. Adjusted Cox models included demographics (age, sex, and BMI), chronic comorbidities (COPD, diabetes, immunosuppression, and chronic liver disease), and study epoch (2011-2016, 2017-2019, and 2020-2024) as a categorical variable to account for potential temporal trends in cohort composition and clinical practice. We prioritized baseline characteristics over acute severity markers based on evidence that chronic health conditions predominantly determine long-term post-ICU outcomes.^19,20^ Additionally, acute physiologic severity parameters are partially inherent to ARF subtype definitions and adjusting for them could attenuate the prognostic differences we sought to characterize. The proportional hazards assumption was assessed using Schoenfeld residuals. We report hazard ratios (HRs) with 95% CIs, using ARDS as the reference group to contrast the non-ARDS subtypes. For the subset of patients classified via virtual consensus meetings (epoch 2020-2024), we calculated intraclass correlation coefficients (ICCs) to assess interrater reliability of radiographic assessments and subtype classifications. We did not perform formal sample size calculations or adjustment for multiple comparisons because this was an exploratory observational cohort study including all consecutively enrolled eligible patients, and findings should be considered hypothesis generating. All analyses were performed using R software version 4.4.3 (R Foundation for Statistical Computing). Results are reported in accordance with the Strengthening the Reporting of Observational Studies in Epidemiology guidelines for observational studies.^21^

## Results

### Classification of ARF Subtypes

We analyzed 826 patients who were mechanically ventilated classified into the following ARF subtypes: ARDS (36.1%), ARFA (34.1%), airway control (10.7%), other or multifactorial (7.4%), CHF (6.2%), AoCHRF (3.3%), and AEILD (2.3%) (Table 2). ARDS prevalence varied significantly across study epochs (37%, 20%, and 55% in 2011-2016, 2017-2019, and 2020-2024, respectively; *P* < .001), with the surge during the third epoch attributable to the COVID-19 pandemic (e-Fig 4). We found that classifications into airway control, AEILD, and ARDS subtypes showed the highest agreement rates (92.9%, 87.5%, and 84.5% unanimous agreements among reviewers, respectively), with lower proportions of unanimous agreements for other subtypes, especially CHF and other or multifactorial (e-Fig 2 and Expanded Results in e-Appendix 1). The 7.4% classified as other or multifactorial reflected genuine diagnostic complexity, including patients with overlapping features across multiple categories or atypical presentations not fitting predefined subtypes.

### Demographics and Clinical Characteristics

ARF subtypes demonstrated distinct baseline and comorbidity profiles (Table 2). Patients receiving airway control were youngest (median, 52.9 years), whereas patients with CHF were oldest (65.0 years). Patients with ARDS and CHF showed higher BMI (31.5 and 32.4 kg/m^2^, respectively) compared with those receiving airway control (27.7 kg/m^2^). Comorbidity patterns reflected underlying disease processes: chronic kidney disease was prevalent in CHF (37.3%), immunosuppression was prevalent in AEILD (31.6%) and ARDS (26.8%), and COPD was prevalent in AoCHRF (66.7%). Pneumonia was the predominant risk factor in patients with ARDS (76.8%) and ARFA (51.8%), whereas nonpulmonary sepsis was more common in patients with ARFA (34.8%) than patients with ARDS (15.4%).

### Applied Treatments and Organ Support

ARDS, CHF, and ARFA showed the highest severity of illness (median modified Sequential Organ Failure Assessment score of 7-8 vs 4-6 in other subtypes). Ventilator management strategies reflected underlying pathophysiologic features (Table 2, e-Fig 5). Patients with AoCHRF received larger tidal volumes (7.4 mL/kg), whereas patients with ARDS and AEILD received higher positive end-expiratory pressure (10.0 and 8.0 cm H_2_O, respectively) and lower tidal volumes (6.4-6.5 mL/kg) (e-Fig 5). Vasopressor requirements were common across most subtypes (67%-89%), with longer duration in ARDS (median, 4 days) compared with other groups (median, 1-3 days). Advanced organ support therapies varied markedly by subtype (e-Fig 6). Continuous renal replacement therapy was most frequent in patients with ARDS (26.2%) and least in patients with AoCHRF (0%). Neuromuscular blockade was used primarily for severe ARDS (43.3%) and AEILD (42.1%). Extracorporeal membrane oxygenation was used in 10% to 12% of patients with ARDS and AEILD for refractory hypoxemia, with minimal use in other subtypes.

### Gas Exchange and Respiratory Mechanics

Gas exchange and respiratory mechanics differed markedly across ARF subtypes, reflecting distinct pathophysiologic processes (e-Fig 7, Table 2). Patients with ARDS and AEILD exhibited the most severe hypoxemia (median Pao_2_ to Fio_2_ ratio, 170 and 162 mm Hg, respectively), whereas patients with airway control maintained near-normal oxygenation (277 mm Hg). Ventilatory ratio—reflecting dead space ventilation—was elevated in patients with AoCHRF (1.9), ARDS (1.9), and AEILD (2.2) compared with those with airway control (1.4). Patients with AEILD demonstrated the highest normalized respiratory system elastance (3.3 vs 1.6-2.2 in other subtypes), consistent with severely reduced lung compliance in fibrotic lung disease. Mechanical power delivered by the ventilator was greatest in ARDS and AEILD (17-20 J/min), reflecting the combination of high driving pressures, respiratory rates, and minute ventilation required to maintain gas exchange in these high-severity subtypes.

### Imaging Characteristics

Patients with ARDS and AEILD showed the highest radiographic lung edema severity (Radiographic Assessment of Lung Edema scores), followed by patients with CHF, whereas patients with airway control showed minimal parenchymal opacities (e-Fig 8). Echocardiographic data (available in 51.6% of patients) demonstrated that patients with CHF harbored significantly reduced left ventricular ejection fraction (median, 37.5% vs 57.5% in patients with ARDS; *P* < .01) and larger cardiac chamber dimensions, with no significant differences among other subtypes (e-Fig 9).

### Biological Profiles and Host-Response Subphenotypes

Plasma biomarker profiles were available for 90.2% of participants, with no significant differences in baseline characteristics between those with and without complete data (e-Table 4). Biomarker concentrations differed dramatically across ARF subtypes (Fig 1, e-Fig 10, Table 2, e-Table 5). Patients with ARDS showed the highest IL-6, Ang-2, and soluble receptor for advanced glycation end-products levels, followed by patients with ARFA and CHF. In contrast, patients with AEILD, airway control, and AoCHRF showed the lowest biomarker levels across subtypes, whereas the other or multifactorial group showed biomarker levels in an intermediate range close to the cohort median (Fig 1A). Hyperinflammatory subphenotype prevalence mirrored biomarker enrichment patterns, ranging from 0% (AoCHRF) to 30.1 (ARFA): ARDS, 25.8%; CHF, 18.8%; other, 15.4%; airway control, 9.1%; and AEILD, 5.6% (Fig 1B).

### Clinical Outcomes

ARF subtypes demonstrated markedly divergent survival trajectories from acute hospitalization through long-term follow-up (Fig 2). During the index admission, patients with ARDS required the longest duration of IMV (median, 10 days) (e-Fig 11). Discharge destinations also varied by subtype, reflecting differences in post-ICU functional independence (e-Fig 12). Patients with airway control (82.4%) and AoCHRF (76.5%) were most frequently discharged to settings reflecting higher independence—home, inpatient psychiatry, or rehabilitation—whereas patients with ARDS most often required ongoing care in long-term acute care hospitals (18.8%) or skilled nursing facilities (20.4%).

Three-year survival varied markedly across ARF subtypes, ranging from 10.5% in AEILD and 37.7% in the other or multifactorial group to 48.1% in patients with AoCHRF, 51.3% in patients with ARDS, 53.2% in patients with ARFA, 56.9% in patients with CHF, and 67.0% in patients with airway control (*P* < .0001, log-rank test) (Fig 2). Overall 3-year survival remained stable across study epochs despite temporal variation in subtype prevalence (e-Fig 13). Notably, patients with AEILD exhibited precipitous early mortality within the first 30 days. The other or multifactorial group showed progressive late mortality, ultimately becoming the second-worst performing subtype after patients with AEILD by 3 years (37.7% survival). In adjusted Cox proportional hazards regression accounting for age, sex, BMI, comorbidities, and study epoch (Table 3, e-Fig 14), the ARF subtype remained a strong independent predictor of 3-year survival. Compared with patients with ARDS (reference), patients with AEILD showed a nearly 3-fold higher hazard of death (adjusted HR, 2.89; 95% CI, 1.73-4.84; *P* < .001), whereas patients with airway control showed a 43% lower hazard (adjusted HR, 0.57; 95% CI, 0.37-0.86; *P* = .007) and patients with ARFA showed a 24% lower hazard (adjusted HR, 0.76; 95% CI, 0.59-0.98; *P* = .03). Notably, use of life support measures during acute hospitalization, such as vasopressors, neuromuscular blockade, and continuous renal replacement therapy, predicted early 90-day mortality, but did not discriminate survival among hospital survivors between those who received and did not receive such therapies (e-Fig 15).

Baseline hyperinflammatory subphenotype was associated strongly with reduced 3-year survival (Fig 3A). Overall, patients with hyperinflammatory showed 12 percentage points’ lower survival compared with patients with hypoinflammation (42.9% vs 54.9%; *P* = .002, log-rank test), with an adjusted HR of 1.52 (95% CI, 1.20-1.93; *P* < .001) after accounting for age, sex, BMI, comorbidities, and study epoch. However, the prognostic impact of subphenotype varied markedly by clinical ARF subtype (Fig 3B). In the ARDS group, patients with hyperinflammation showed a 16 percentage points’ lower 3-year survival compared with patients with hypoinflammation (39.7% vs 56.1%; *P* = .02). Similarly, in ARFA, patients with hyperinflammation showed 14 percentage points’ lower survival (42.9% vs 57.0%; *P* = .04). In contrast, subphenotype was not associated significantly with survival in other ARF subtypes (*P* > .05 for all).

## Discussion

In this prospective cohort of 826 mechanically ventilated adults with ARF followed up through 3 years, we found that although 70.5% of patients survived to hospital discharge, 51.9% of patients remained alive at 3 years. Importantly, 3-year survival varied markedly across ARF subtypes, ranging from 10.5% in patients with AEILD to 67.0% in patients receiving airway control. ARF subtypes also demonstrated heterogeneous clinical presentations, physiologic derangements, and biological profiles. Prognostic differences persisted after adjustment for age and comorbidities and were reflected in distinct patterns of discharge destinations, with patients receiving airway control and patients with AoCHRF achieving high rates of functional independence, whereas survivors of ARDS commonly required prolonged postacute care. Critically, the hyperinflammatory subphenotype—which predicted worse 3-year survival particularly among patients with ARDS and ARFA—varied dramatically in prevalence across clinical subtypes, demonstrating that clinical and biological classifications capture complementary, rather than redundant, prognostic information.

Our findings highlight a major gap in evidence for non-ARDS ARF.^3,8,10,15,22^ Most ventilated patients did not meet ARDS criteria and displayed considerable heterogeneity in risk factors, physiologic features, management, and outcomes. Patients with ARFA showed similar 3-year survival to those with ARDS despite not meeting full diagnostic criteria. Notably, patients with CHF exhibited a paradoxical profile: elevated epithelial and endothelial injury biomarkers comparable with patients with ARDS, yet shorter duration of mechanical ventilation, better functional recovery at discharge, and similar long-term survival. The presence of hyperinflammatory subphenotype in a minority of patients with CHF (18.8%) may reflect cardiogenic shock with concurrent systemic inflammation, analogous to the hyperinflammatory endotype recently described in cardiogenic shock characterized by worse hemodynamics and higher mortality.^23,24^ This biological similarity to ARDS despite divergent short-term trajectories warrants dedicated investigation into ARF presentations driven by hydrostatic edema mechanisms yet exhibiting systemic inflammation and endothelial-epithelial injury.

AEILD represents a pathophysiologically and clinically distinct ARF entity that warrants particular recognition. Patients with AEILD—defined by acute-on-chronic hypoxemia and diffuse ground-glass opacities superimposed on underlying pulmonary fibrosis—occasionally may fulfill Berlin clinical criteria for ARDS, and prior autopsy series have reported diffuse alveolar damage patterns in some patients.^16^ However, our findings demonstrated marked biological and prognostic dissociation from ARDS. Patients with AEILD exhibited exceptionally poor survival (10.5% at 3 years). Patients with AEILD showed severely reduced respiratory compliance and refractory hypoxemia, yet paradoxically low systemic inflammatory biomarker levels and minimal hyperinflammatory subphenotype prevalence (5.6%). This biological profile—severe acute lung injury with minimal systemic inflammation—contrasts sharply with ARDS and suggests fundamentally different underlying pathophysiologic characteristics. Existing literature on AEILD requiring mechanical ventilation reports in-hospital mortality of 50% to 80%,^25,26^ and our findings extend these observations, demonstrating exceptionally poor prognosis through 3 years. Although patients with AEILD often are excluded from ARDS clinical trials through criteria excluding advanced underlying lung disease, our data underscore the need for dedicated AEILD research. The dismal prognosis, distinct biological features, and increasing prevalence of interstitial lung disease highlight AEILD as a critical target for therapeutic development and outcome prediction.

Long-term outcome data through 3 years highlight the functional impact of ARF subtypes beyond survival to hospital discharge alone. Only 44% of survivors of ARDS achieved independent discharge to home care, whereas patients receiving airway control and patients with AoCHRF subtypes showed markedly better functional recovery. Use of intensive therapies such as neuromuscular blockade and continuous renal replacement therapy was associated strongly with early mortality, but not with long-term survival, among hospital survivors (e-Fig 6), suggesting that initial severity requiring these interventions does not necessarily portend worse outcomes among those who survive to discharge.

Biological subphenotypes provided prognostic information complementary to clinical classification. The hyperinflammatory subphenotype was associated with reduced 3-year survival overall (adjusted HR, 1.52; *P* < .001), but this effect was concentrated in ARDS and ARFA subtypes, where hypoinflammatory patients demonstrated substantially better outcomes. In contrast, subphenotypes were not associated significantly with survival in other ARF subtypes, likely reflecting both limited statistical power—given smaller sample sizes and lower hyperinflammatory prevalence (0%-19% in non-ARDS and non-ARFA subtypes)—and likely diminished prognostic relevance where clinical syndrome predominates. This differential prognostic impact suggests that precision critical care requires integration of both biological and clinical classification: subphenotype-directed interventions may benefit populations with ARDS and ARFA, whereas etiologic-specific management remains paramount in other ARF presentations. Because point-of-care biomarker platforms enable real-time subphenotyping,^27^ combining biological subphenotypes with clinical subtypes can support targeted prognostication and therapeutic decision-making across the heterogeneous spectrum of ARF.

This work presents a pragmatic and reproducible framework with explicit operational definitions for retrospective consensus classification of ARF. Our approach assigns subtypes as “destination” classifications based on the most severe presentation during early critical illness, rather than tracking temporal trajectories of disease evolution from symptom onset to enrollment. Because we focused exclusively on patients who were mechanically ventilated, most already had expressed a definitive ARF phenotype (ARDS vs non-ARDS) by the time of intubation and study enrollment.^28^ The recent global ARDS definition,^7^ which includes patients receiving high-flow nasal cannula, creates opportunities for earlier ARDS identification and subphenotype-directed interventions in patients who were not intubated. However, applying classification frameworks to high-flow nasal cannula oxygenation-supported patients faces unique challenges: dynamic support levels, variable FIO_2_ documentation, and distinguishing transient from established ARDS. We previously demonstrated that plasma biomarkers have prognostic value in distinguishing high-flow nasal cannula oxygenation-supported patients who will avoid intubation in a COVID-19 cohort.^18^ Our IMV-focused approach provides a complementary framework for patients with advanced ARF, where subtypes typically have declared themselves. We recognize that our approach is limited by the scope of available clinical data and depth of diagnostic workup. Certain categories—such as the 7.4% classified as other or multifactorial—highlight diagnostic uncertainty and illustrate limitations of routine clinical assessment.^29^ These populations, exemplified by complex patients such as transplant recipients with undifferentiated ARF where infection, rejection, and pulmonary edema are all plausible, suggest the need for deeper diagnostic investigations, including advanced imaging, lower respiratory tract sampling, and molecular diagnostics,^30^ to characterize and classify complex ARF presentations better.^31^ The poor long-term survival of this diagnostically heterogeneous group (37.7% at 3 years) highlights an important opportunity to refine clinical subtype classification through more intensive diagnostic evaluation.

Our study has several important limitations. The single-center design and long enrollment period (2011-2024) spanning evolving critical care practices limit generalizability, particularly to trauma, surgical, or neurologic ICU populations not well represented in this predominantly medical-cardiac ICU cohort. We lacked independent external validation. The distribution of ARF subtypes does not reflect true ICU population prevalence: although formal inclusion criteria remained unchanged, enrollment priorities evolved from an initial focus on ARDS and ARFA toward broader ARF definitions as collaborator interests and logistical capacity expanded. Additionally, ARDS prevalence surged during the COVID-19 pandemic (55% in 2020-2024 vs 20%-37% in earlier epochs) (e-Fig 4). Therefore, subtype proportions in the present cohort reflect enrollment patterns and pandemic effects, rather than baseline epidemiologic prevalence. Vital statistics for survival ascertainment were obtained through February 2025, which limited follow-up for more recently enrolled participants. However, 94% of participants were enrolled before 2023. Several ARF subtypes had small sample sizes yielding underpowered analyses with wide CIs. The absence of significant findings for such subtypes may reflect insufficient statistical power, rather than true null effects. We performed multiple comparisons without adjustment for multiplicity, and findings should be considered exploratory and hypothesis-generating, requiring validation in larger multicenter cohorts. We did not capture pulmonary embolism diagnoses or cognitive, physical, and psychological outcomes after discharge systematically, important domains of recovery after critical illness.^32,33^ Consensus clinical classifications were performed retrospectively after study completion, and biological subphenotypes were defined using batch research laboratory measurements, rather than point-of-care assays, precluding real-time subphenotype assessments. However, the Phenotypes in Acute Respiratory Distress Syndrome (PHIND) trial recently demonstrated feasibility of bedside biological subphenotyping,^27^ providing a path forward for prospective subphenotype-based care. Despite these limitations, our systematic subtyping revealed marked prognostic and management differences, supporting its value for guiding future multicenter studies and the development of subtype-specific interventions.

## Interpretation

Mechanically ventilated adults with ARF were shown to demonstrate marked heterogeneity in presentation, biological characteristics, and long-term outcomes. Although clinicians routinely differentiate ARF causes in practice, our systematic framework provides reproducible research standards with explicit operational definitions and quantitative long-term outcomes extending beyond hospital discharge. This enables standardized subtype-stratified enrollment in clinical trials and evidence-based prognostic benchmarks for understudied populations. Critically, biological subphenotypes strongly influenced outcomes in ARDS and ARFA, whereas clinical syndrome predominated in other subtypes, suggesting that precision critical care requires integrating both clinical and biological classification, rather than relying on either alone. Validation in independent multicenter cohorts will be essential to establish generalizability and to refine subtype-specific therapeutic strategies in ARF.

## Supplementary Material

1

2

## Figures and Tables

**Figure 1 –:**
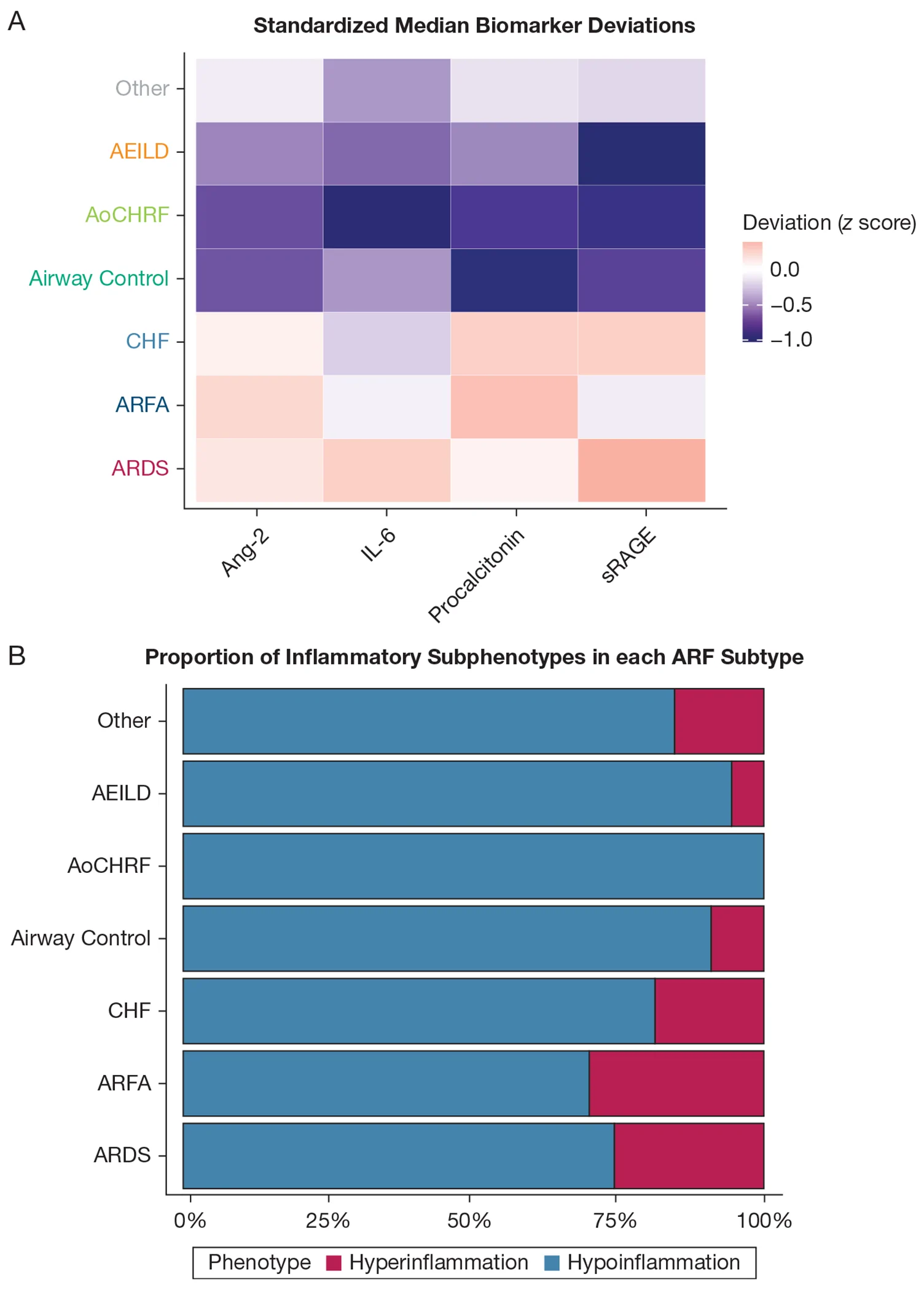
Biological comparisons of ARF clinical subtypes and distribution of host-response subphenotypes. A, Heatmap showing standardized median plasma biomarker deviations (z scores) across ARF subtypes. Each tile represents the median deviation of a biomarker relative to the overall cohort distribution, highlighting biologic clustering of IL-6, procalcitonin, sRAGE, and Ang-2 across subtypes. Patients receiving extracorporeal membrane oxygenation were excluded because of the potential effects of the extracorporeal circuit on biomarker levels (eg, endothelial injury) that may not reflect the primary biological process. B, Normalized stacked bar plots illustrating the proportion of inflammatory subphenotypes (hyperinflammatory vs hypoinflammatory) within each ARF subtype. Bars are scaled to 100% of the subtype population. AEILD = acute exacerbation of interstitial lung disease; Ang-2 = angiopoietin-2; AoCHRF = acute-on-chronic hypercapnic respiratory failure; ARF = acute respiratory failure; ARFA = at risk for ARDS; CHF = congestive heart failure; sRAGE = soluble receptor for advanced glycation end-products.

**Figure 2 –:**
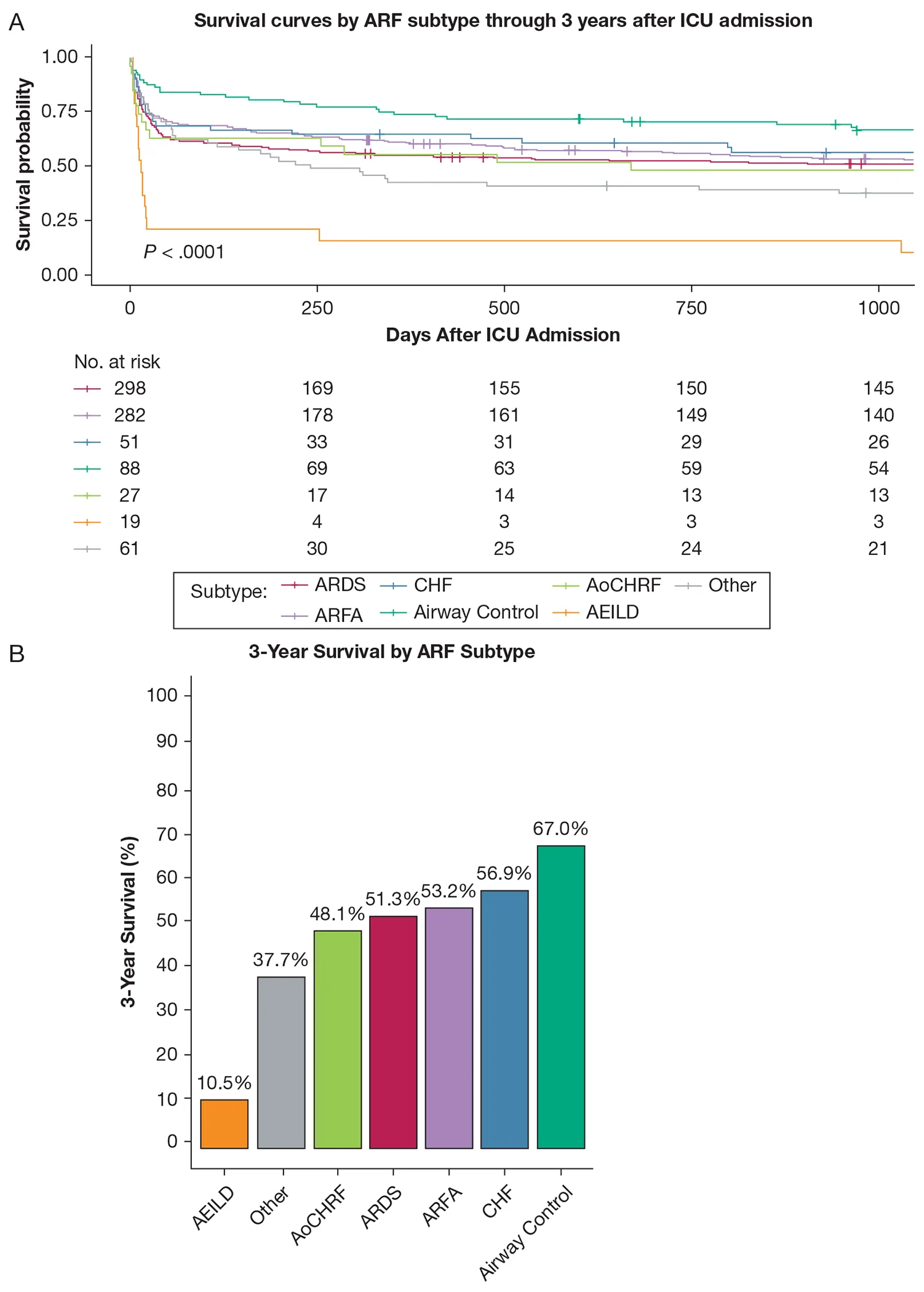
Clinical outcomes by ARF subtype. A, Kaplan-Meier survival curves through 3 years demonstrating marked heterogeneity in mortality across clinical subtypes, with AEILD showing the highest risk of death and patients receiving airway control showing the lowest. B, Bar graph showing 3-year survival by subtype in ascending order, revealing a spectrum of outcomes ranging from 10.5% in AEILD to 67% in patients receiving airway control. AEILD = acute exacerbation of interstitial lung disease; AoCHRF = acute-on-chronic hypercapnic respiratory failure; ARF = acute respiratory failure; ARFA = at risk for ARDS; CHF = congestive heart failure.

**Figure 3 –:**
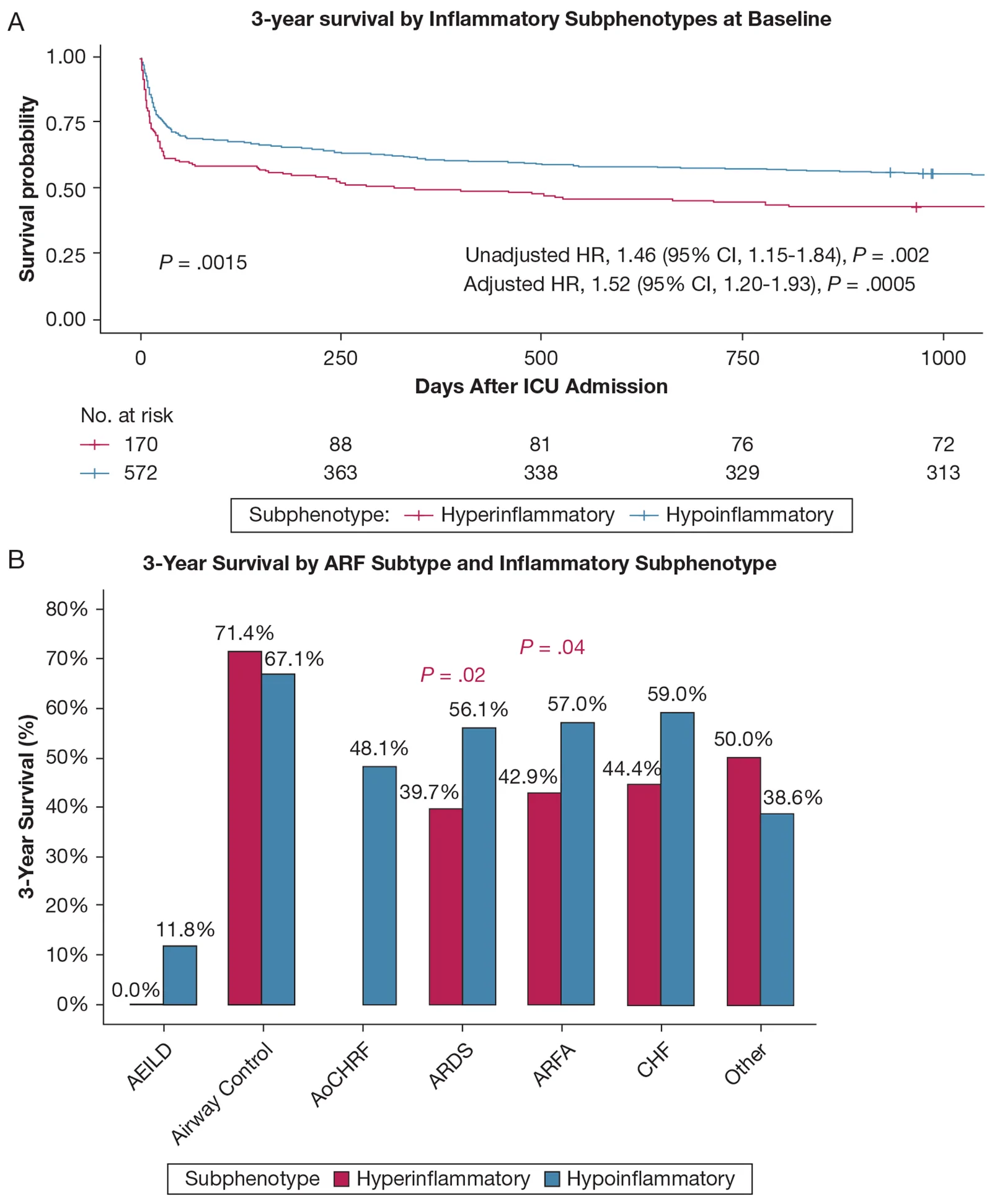
A, B, Long-term survival by inflammatory subphenotypes (A), stratified by clinical subtypes of ARF (B). A, Kaplan-Meier curves for 3-year survival comparing patients with hyperinflammation vs patients with hypoinflammation, based on baseline biomarker measurements. Patients with hyperinflammation demonstrated significantly lower survival (P = .0015, log-rank test). Unadjusted and adjusted HRs with 95% CIs are shown from Cox models estimate of HR for death at 3 years using the hypoinflammatory subphenotype as the reference category. Adjusted models account for age, sex, BMI, history of COPD, diabetes, immunosuppression, chronic liver disease, and study epoch. The proportional hazards assumption was met for both unadjusted (P = .13) and adjusted (P = .21) models. B, Bar graph showing 3-year survival proportions for patients with hyperinflammation and patients with hypoinflammation within ARF subtypes, with significance testing performed using Fisher tests with continuity correction. Significant differences were observed only in the ARDS and ARFA subtypes, in which patients with hypoinflammation showed higher 3-year survival. AEILD = acute exacerbation of interstitial lung disease; AoCHRF = acute-on-chronic hypercapnic respiratory failure; ARF = acute respiratory failure; ARFA = at risk for ARDS; CHF = congestive heart failure; HR = hazard ratio.

**TABLE 1 ]:** Definitions of ARF Subtypes Used for Consensus Classification

Subtype	Operational Definition
ARDS	Berlin definition criteria:
	Timing: onset within 1 week of a known clinical insult or new or worsening respiratory symptoms.
	Chest imaging: bilateral opacities must be observed on chest radiography or CT imaging, not fully explained by effusions, lobar or lung collapse, or nodules.
	Origin of edema: respiratory failure not fully explained by cardiac failure or fluid overload. Objective assessment (eg, echocardiography) should be used to exclude hydrostatic edema if no risk factor is present.
	Oxygenation: Pao_2_ to Fio_2_ ratio of ≤ 300 with PEEP or CPAP of ≥ 5 cm H_2_O.
ARFA	Presence of an identifiable direct or indirect risk factor for lung injury, but not fulfilling chest imaging or oxygenation criteria for ARDS.^7^
CHF	Chest imaging with bilateral opacities consistent with pulmonary edema and objective assessment of cardiac function and volume status supportive of primarily hydrostatic edema mechanism. Cardiac dysfunction could include either systolic or diastolic dysfunction. Patients with pulmonary edema in the presence of risk factors for lung injury (eg, pneumonia) could not be classified as having CHF because the mechanism of edema could be increased permeability (ie, ARDS) and not purely hydrostatic, which was the intended patient population classified in the CHF subtype.
Intubation for airway protection (airway control)	Intubation for airway protection with no evidence of acute lung injury on imaging other than possible atelectasis and no identifiable risk factor for lung injury. Examples included patients intubated for encephalopathy (pharmacologic overdose, stroke, and hepatic encephalopathy) without evidence of macroaspiration or patients with gastrointestinal bleeding intubated for endoscopy.
AoCHRF	Presentation with decompensated hypercapnic respiratory failure, including a Paco_2_ (or Pvco_2_) > 45 mm Hg and pH < 7.35 because of acute exacerbation of obstructive airways disease and without any identifiable risk factor for lung injury.
AEILD	Presentation with acute hypoxemic respiratory failure with diffuse ground-glass opacities on chest imaging and history of ILD. AEILD could be triggered by respiratory infections (direct risk factor for lung injury),^16^ but was deemed as distinct from ARDS.
Other or multifactorial (unable to determine primary cause)	Patients for whom the committee members could not reach consensus on classification into one of the other distinct subtypes of ARF. For example, patients with history of double lung transplantation and hypoxemic respiratory failure with undiagnosed pulmonary opacities who received empiric treatments for pulmonary edema, infection, and allograft rejection.

AEILD = acute exacerbation of interstitial lung disease; AoCHRF = acute-on-chronic hypercapnic respiratory failure; ARF = acute respiratory failure; ARFA = at risk for ARDS; CHF = congestive heart failure; ILD = interstitial lung disease; PEEP = positive end-expiratory pressure; Pvco_2_ = partial pressure of venous CO_2_.

**TABLE 2 ]:** Patient Characteristics by Clinical ARF Subtype

Characteristic	AoCHRF (n = 27)	AEILD (n = 19)	Airway Control (n = 88)	ARDS (n = 298)	ARFA (n = 282)	CHF (n = 51)	Other or Multifactorial (n = 61)
Demographics							
Age, y	60.2 (53.5-67.6)	62.6 (58.5-65.4)	52.9 (41.4-62.7)	56.3 (44.4-66.3)	61.0 (51.2-69.8)	65.0 (51.1-72.2)	60.7 (48.2-68.0)
Male sex	10 (37.0)	13 (68.4)	51 (58.0)	166 (55.7)	168 (59.6)	32 (62.7)	34 (55.7)
BMI, kg/m^2^	27.1 (24.2-32.7)	28.1 (25.8-34.4)	27.7 (24.8-32.2)	31.5 (26.3-36.2)	29.5 (25.1-35.9)	32.4 (27.1-40.0)	28.5 (23.5-35.8)
Comorbidities							
CHF	7 (25.9)	0 (0.0)	6 (6.8)	31 (10.4)	47 (16.7)	12 (23.5)	6 (9.8)
Diabetes mellitus	6 (22.2)	4 (21.1)	22 (25.0)	92 (30.9)	117 (41.5)	21 (41.2)	21 (34.4)
COPD	18 (66.7)	3 (15.8)	9 (10.2)	46 (15.4)	72 (25.5)	10 (19.6)	11 (18.0)
Chronic kidney disease	1 (3.7)	0 (0.0)	12 (13.6)	37 (12.4)	49 (17.4)	19 (37.3)	16 (26.2)
Immunosuppression	8 (29.6)	6 (31.6)	10 (11.4)	80 (26.8)	40 (14.2)	12 (23.5)	18 (29.5)
Chronic liver disease	2 (7.4)	1 (5.3)	15 (17.0)	30 (10.1)	33 (11.7)	1 (2.0)	10 (16.4)
Pulmonary fibrosis	1 (3.7)	18 (94.7)	0 (0.0)	8 (2.7)	1 (0.4)	0 (0.0)	5 (8.2)
Solid organ transplant	1 (3.7)	0 (0.0)	4 (4.5)	45 (15.1)	16 (5.7)	7 (13.7)	15 (24.6)
Lung transplantation	1 (3.7)	0 (0.0)	0 (0.0)	21 (7.0)	7 (2.5)	1 (2.0)	11 (18.0)
Vital signs							
Temperature, °C	37.1 (36.2-37.7)	36.6 (36.2-36.8)	36.6 (36.0-37.2)	36.9 (36.4-37.4)	36.9 (36.3-37.6)	37.0 (36.4-37.5)	36.6 (36.0-37.1)
Heart rate, beats/min	83 (76-100)	88 (79-97)	90 (77-98)	89 (76-103)	91 (78-105)	91 (80-101)	85 (72-95)
Respiratory rate, breaths/min	16 (14-25)	24 (23-30)	18 (15-21)	23 (18-27)	20 (16-24)	19 (17-22)	18 (16-22)
Systolic BP, mm Hg	120 (102-132)	110 (101-117)	126 (106-142)	114 (102-128)	118 (102-134)	118 (107-132)	119 (106-139)
Oxygen saturation, %	98 (96-100)	96 (94-98)	99 (97-100)	96 (94-98)	98 (95-100)	98 (96-99)	98 (95-99)
Ventilatory parameters							
Tidal volume, mL/kg	7.4 (6.5-8.1)	6.4 (5.2-7.0)	6.7 (6.0-7.6)	6.5 (6.0-7.5)	6.8 (6.1-7.8)	6.7 (6.0-7.7)	6.8 (6.0-8.0)
Minute ventilation, L/min	7.6 (6.0-10.8)	11.5 (10.2-13.1)	8.7 (7.0-10.7)	9.9 (8.0-12.0)	9.5 (7.6-11.3)	8.6 (7.3-10.5)	8.4 (7.3-9.8)
PEEP, cm H_2_O	5.0 (5.0-5.0)	8.0 (5.5-10.0)	5.0 (5.0-5.0)	10.0 (6.0-12.0)	5.0 (5.0-8.0)	5.3 (5.0-9.0)	5.0 (5.0-8.0)
Plateau pressure, cm H_2_O	23 (17-25)	30 (23-32)	16 (13-18)	25 (21-29)	18 (15-23)	20 (17-25)	20 (16-25)
Normalized elastance	2.0 (1.6-2.8)	3.3 (2.0-4.2)	1.6 (1.2-2.0)	2.2 (1.6-2.9)	1.7 (1.3-2.1)	1.8 (1.4-2.2)	1.9 (1.5-2.5)
Ventilatory ratio	1.9 (1.5-2.5)	2.2 (1.9-2.8)	1.4 (1.2-1.7)	1.9 (1.5-2.3)	1.5 (1.1-1.8)	1.5 (1.2-1.8)	1.6 (1.3-1.8)
Pao_2_ to Fio_2_ ratio	196 (138-280)	162 (133-194)	277 (232-284)	170 (121-230)	243 (181-284)	240 (212-295)	218 (163-268)
Clinical laboratory values							
WBC count, ×10^9^/L	10.5 (8.0-14.8)	11.4 (8.2-14.9)	10.3 (8.0-14.1)	12.3 (9.0-18.2)	12.7 (9.3-17.1)	11.1 (7.9-16.2)	10.8 (6.9-14.9)
Hemoglobin, g/dL	11.3 (10.2-12.8)	11.0 (9.4-11.7)	11.4 (10.0-13.2)	10.1 (8.7-11.8)	10.6 (9.2-12.4)	10.9 (9.4-12.7)	10.4 (8.6-11.6)
Platelets, ×10^9^/L	215 (182-250)	214 (166-274)	177 (123-239)	192 (122-265)	181 (128-242)	176 (131-230)	177 (136-220)
Creatinine, mg/dL	0.9 (0.7-1.4)	0.8 (0.7-0.9)	0.9 (0.6-1.2)	1.2 (0.8-2.3)	1.3 (0.8-2.4)	2.0 (1.1-3.2)	1.5 (0.9-2.7)
Bicarbonate, mEq/L	33 (26-35)	28 (26-33)	23 (21-25)	25 (22-28)	23 (20-26)	25 (22-29)	25 (22-29)
Plasma biomarkers, pg/mL							
IL-6	11.8 (6.2-35.5)	24.9 (8.5-63.3)	35.6 (19.1-69.4)	117.9 (33.4-379.1)	65.2 (23.7-194.7)	52.3 (26.0-121.4)	34.6 (18.1-71.7)
IL-8	12.3 (6.0-21.7)	18.0 (8.5-28.4)	8.7 (5.4-20.0)	24.2 (14.4-44.8)	20.3 (9.4-40.4)	12.6 (7.7-22.8)	13.6 (7.2-24.2)
sTNFR1	2,376 (1,662-3,215)	2,279 (1,701-3,087)	2,042 (1,353-4,008)	4,946 (3,085-8,845)	4,520 (2,632-8,157)	5,485 (2,989-9,410)	4,391 (2,629-8,172)
sRAGE	1,573 (1,008-2,707)	1,124 (353-2,234)	1,781 (1,258-2,723)	5,275 (2,609-9,811)	3,629 (2,211-6,359)	4,737 (3,261-8,005)	3,264 (2,145-6,163)
Angiopoietin-2	3,451 (2,242-8,261)	3,932 (2,678-6,786)	3,551 (2,070-5,911)	7,609 (3,915-15,308)	8,463 (4,079-17,579)	7,566 (4,640-14,825)	6,382 (4,116-12,261)
Procalcitonin	194.8 (73.7-638.3)	353.7 (142.4-751.2)	159.3 (70.3-581.6)	745.6 (309.4-2966.2)	1177.2 (294.6-4040.7)	1038.2 (384.5-2792.2)	599.9 (184.1-1761.3)
Severity of illness							
mSOFA score	6.0 (3.8-7.2)	6.0 (4.0-9.2)	4.0 (3.0-7.0)	8.0 (5.0-10.0)	7.0 (5.0-9.8)	8.0 (5.5-10.0)	6.5 (5.0-9.0)
Risk factors for ARDS							
Pneumonia	1 (3.7)	6 (31.6)	1 (1.1)	229 (76.8)	146 (51.8)	0 (0.0)	6 (9.8)
Nonpulmonary sepsis	0 (0.0)	2 (10.5)	0 (0.0)	46 (15.4)	98 (34.8)	1 (2.0)	4 (6.6)
Aspiration	0 (0.0)	2 (10.5)	1 (1.1)	36 (12.1)	68 (24.1)	0 (0.0)	4 (6.6)
COVID-19	0 (0.0)	0 (0.0)	0 (0.0)	98 (32.9)	26 (9.2)	0 (0.0)	4 (6.6)
Host-response subphenotype							
Hyperinflammatory^a^	0 (0.0)	1 (5.6)	8 (9.1)	68 (25.8)	77 (30.1)	9 (18.8)	8 (15.4)
Clinical outcomes							
Ventilator-free days	20 (5-26)	0 (0-22)	25 (21-27)	11 (0-21)	21 (7-25)	25 (17-26)	21 (8-25)
Duration of MV, d	5.0 (2.0-8.0)	7.0 (5.5-11.5)	3.0 (1.0-6.0)	10.0 (5.0-19.0)	6.0 (3.0-11.0)	3.0 (2.0-6.0)	5.0 (3.0-11.0)
90-d survival	17 (63.0)	4 (21.1)	74 (84.1)	184 (61.7)	194 (68.8)	35 (68.6)	38 (62.3)
3-y survival	13 (48.1)	2 (10.5)	59 (67.0)	153 (51.3)	150 (53.2)	29 (56.9)	23 (37.7)

Data are presented as No. (%) or median (interquartile range). AEILD = acute exacerbation of interstitial lung disease; AoCHRF = acute-on-chronic hypercapnic respiratory failure; ARFA = at risk for ARDS; CHF = congestive heart failure; mSOFA = modified Sequential Organ Failure Assessment; MV = mechanical ventilation; PEEP = positive end-expiratory pressure; sRAGE = soluble receptor for advanced glycation end-products; sTNFR1 = soluble tumor necrosis factor receptor-1.

a Phenotype determined using the 4-biomarker parsimonious model (procalcitonin, angiopoietin-2, sTNFR1, bicarbonate). Phenotype data available for 745 of 826 participants (90.2%).

**TABLE 3 ]:** Cox Proportional Hazards Models Performed to Estimate Hazard Ratios for Death at 3 Years Using ARDS as the Reference Category

ARF Subtype	Unadjusted HR (95% CI)	*P* Value^a^	Adjusted HR (95% CI)	*P* Value^a^
ARDS (reference)	1.00	NA	1.00	NA
ARFA	0.91 (0.72-1.16)	.451	**0.76 (0.59-0.98)**	**.032**
CHF	0.85 (0.54-1.32)	.464	0.69 (0.44-1.09)	.114
Airway control	**0.56 (0.38-0.84)**	**.005**	**0.57 (0.37-0.86)**	**.007**
AoCHRF	1.12 (0.64-1.93)	.697	1.12 (0.63-1.99)	.708
AEILD	**3.27 (1.97-5.41)**	**< .001**	**2.89 (1.73-4.84)**	**< .001**
Other or multifactorial	1.32 (0.93-1.89)	.125	1.16 (0.81-1.68)	.418

Adjusted models account for age; sex; BMI; history of COPD, diabetes, immunosuppression, and chronic liver disease; and study epoch. Boldface values indicate significant results (*P* < .05). AEILD = acute exacerbation of interstitial lung disease; AoCHRF = acute-on-chronic hypercapnic respiratory failure; ARF = acute respiratory failure; ARFA = at risk for ARDS; CHF = congestive heart failure; HR = hazard ratio; NA = not applicable.

a Cox regression. Proportional hazards assumption was met for both unadjusted (*P* = .25) and adjusted (*P* = .23) models.

## Take-Home Points

### Study Question:

How do clinical subtypes of acute respiratory failure (ARF) requiring mechanical ventilation differ in physiologic characteristics, biological profiles, and long-term survival?

### Results:

Among 826 mechanically ventilated adults followed up for 3 years, overall survival was 51.9%, but varied markedly by clinical subtype: from 10.5% in acute exacerbation of interstitial lung disease to 67.0% in patients receiving airway control. Hyperinflammatory phenotype was associated with lower 3-year survival (42.9% vs 54.9%; *P* < .01), with the strongest effect observed in ARDS and ARFA subtypes, whereas other subtypes showed minimal phenotype-associated survival differences.

### Interpretation:

Our results show that clinical subtype classification identifies distinct populations with ARF with divergent long-term prognoses beyond traditional ARDS categorization. Integration of clinical subtypes with biological phenotyping was shown to provide complementary prognostic information to guide risk stratification, to inform prognostic counseling, and to support precision critical care approaches in patients who are mechanically ventilated.

## ABBREVIATIONS:

AEILD: acute exacerbation of interstitial lung disease
Ang-2: angiopoietin-2
AoCHRF: acute-on-chronic hypercapnic respiratory failure
ARF: acute respiratory failure
ARFA: at risk for ARDS
CHF: congestive heart failure
HR: hazard ratio
IMV: invasive mechanical ventilation
