# Supplementary material for: Characterization and 3-Year Survival of Clinical Subtypes and Host Response Phenotypes of Acute Respiratory Failure Requiring Mechanical Ventilation
Source: CHEST Crit Care. Author manuscript; Available in PMC 2026 Oct 1. (PMC13626120; doi:10.1016/j.chstcc.2026.100274)
Supplement: Supplementary file 1 [file NIHMS2210626-supplement-1.docx]

**Supplement**

**Study Design and Population:**

We conducted a prospective cohort study of adults with acute respiratory failure (ARF) on invasive mechanical ventilation (IMV), enrolled in a clinical registry and biospecimen repository in a tertiary academic center from October 2011 through April 2024.[^1–7^](https://sciwheel.com/work/citation?ids=17902524,7657922,10433421,12208454,15886182,14818913,16531108&pre=&pre=&pre=&pre=&pre=&pre=&pre=&suf=&suf=&suf=&suf=&suf=&suf=&suf=&sa=0,0,0,0,0,0,0&dbf=0&dbf=0&dbf=0&dbf=0&dbf=0&dbf=0&dbf=0) The study was approved by the University of Pittsburgh IRB and informed consent was obtained from patients or their legally authorized representatives. All procedures followed the ethical standards of the Declaration of Helsinki. Eligible patients for this cohort were adults (18-90 years) admitted to intensive care units (ICUs) and requiring IMV via endotracheal intubation for ARF from diverse etiologies including pneumonia, sepsis, aspiration, cardiogenic pulmonary edema, chronic lung disease exacerbations, and airway protection needs. We excluded patients with chronic ventilator dependence via tracheostomy.

Patients were enrolled from five ICUs across three UPMC hospitals: Presbyterian MICU (32-bed academic medical ICU, 85.2% enrollment), Presbyterian Cardiac ICU (8-bed, 5.9%), Presbyterian Transplant/Trauma ICUs (22-bed academic ICUs 1.7%), UPMC East general ICU (16-bed community hospital, 3.7%), and Shadyside Medical ICU (21-bed with high oncology case mix, 3.2%).

This study employed a prospective observational cohort design with retrospective clinical adjudications.

Prospective Components:

- Enrollment: Participants were enrolled prospectively after obtaining informed consent from patients or legally authorized representatives
- Biospecimen Collection: Plasma samples were collected prospectively at predefined timepoints (baseline within 72 hours of intubation, middle follow-up at days 3-6, late follow-up at days 7-10)
- Clinical Data Collection: Demographics, comorbidities, vital signs, ventilatory parameters, and laboratory values were extracted from the electronic medical record during the acute hospitalization
- Outcome Tracking: Long-term survival was tracked prospectively through medical record review and linkage with vital statistics databases

Retrospective Components:

- Clinical subtype adjudication: Consensus classification of ARF subtypes was performed retrospectively after participants completed all study procedures. This allowed the adjudication committee to review the complete clinical course, including evolution of respiratory failure over the first 10 days, and diagnostic findings, rather than making real-time classifications based on incomplete information. Committee members remained blinded to biomarker results and long-term outcomes during adjudication.
- Biomarker Measurements: Plasma biomarkers were measured in batches rather than in real-time to optimize laboratory and cost efficiency, reduce inter-assay variability, and enable consistent quality control across the entire cohort.

**Consensus-based classifications:**

Our consensus classification approach evolved in response to external logistical factors and emerging literature, distinguishing two temporally independent phases in our approach:

*Summary discussion consensus* (Pre-COVID-19): From cohort inception through March 2020 (onset of COVID-19 pandemic), we held in-person meetings to review all available clinical data for participants who had completed study procedures (see below). A study coordinator prepared a brief written summary of each case, which was then reviewed along with chest imaging and electronic medical record (EMR) data by a committee of board-certified critical care physician scientists. Committee members were blinded to both the research biomarkers and patient outcomes at the time of the meeting. An open discussion followed to assign participants to one of seven predefined ARF subtypes (Table 1), which were selected for their face and construct validity in representing the range of ARF presentations:

1. **ARDS** per Berlin definition criteria.[^8^](https://sciwheel.com/work/citation?ids=585910&pre=&suf=&sa=0&dbf=0)
2. **At-risk for ARDS (ARFA)**: Identifiable risk factor(s) for acute lung injury (e-**Table 2**) but not fulfilling ARDS diagnostic criteria.[^2,9^](https://sciwheel.com/work/citation?ids=7657922,12208446&pre=&pre=&suf=&suf=&sa=0,0&dbf=0&dbf=0)
3. **Congestive Heart Failure (CHF)**: hypoxemic ARF from cardiogenic pulmonary edema.
4. **Airway Controls**: intubation for airway protection without primary pulmonary pathology.
5. **Acute-on-Chronic Hypercapnic Respiratory Failure (**AoCHRF**)**: Acute decompensation of chronic ventilatory failure from obstructive airways disease.
6. **Acute Exacerbation of Interstitial Lung Disease (AEILD)**: Acute worsening of ILD with diffuse radiographic abnormalities.[^10^](https://sciwheel.com/work/citation?ids=1837087&pre=&suf=&sa=0&dbf=0)
7. **Other/Multifactorial**: Patients not clearly fitting other categories or with overlapping features from multiple subtypes.

For each participant, subtype classification was first determined retrospectively for the available data upon study enrollment. Committee members voted on the appropriate ARF subtype classification, with the majority vote determining the final classification. Notably, we did not capture inter-rater agreement data during these summary consensus meetings.

b) *Survey-based consensus*: In-person consensus meetings were suspended from March 2020 through September 2020 due to the COVID-19 pandemic response. From October 2020 through April 2024, we transitioned to a secure virtual platform (via institutional Microsoft Teams accounts) to continue our consensus process. The methodology was adapted to include a standardized review of clinical data in a predefined sequence: radiographic, gas exchange, clinical history, physiology/laboratory, microbiology, and cardiovascular data (e-Figure 1 and Supplemental File 1). This review was conducted anonymous voting using an online platform. Prior to the virtual meetings, a study investigator (GDK) performed an in-depth review of each case. Subsequently, a study coordinator prepared a standardized PowerPoint presentation of the clinical data in a consistent order, ensuring clarity and uniformity across case presentations (Supplemental File 1). At the first virtual session, GDK conducted a one-hour training session for all committee members on the operational definitions used in this process, focusing on ARDS/ARF diagnosis, radiographic assessments, and key study methodology. Additionally, new members were provided with relevant literature on ARDS/ARF diagnosis to ensure all participants had a solid understanding of the clinical context.[^8,11^](https://sciwheel.com/work/citation?ids=585910,1761451&pre=&pre=&suf=&suf=&sa=0,0&dbf=0&dbf=0) Committee members were instructed to base their votes only on the available data at the time of each assessment. No clinical outcome data were presented during voting to avoid potential bias. Committee members could request clarifications and review additional EMR data as needed, but clinical outcome data were intentionally withheld.

At each time point (baseline [day 1], middle [days 3-6], or late follow up [7-10]), committee members responded to three multiple-choice questions:

*1) Is this radiographic presentation consistent with ARDS?*

*2) What is the appropriate ARF subtype for this subject?*

*3) Does this subject have risk factors for lung injury? (select as many appropriate),*

Radiographic assessments were made first, before any additional clinical details were presented. Reviewers were asked to rate the radiographic findings as "yes" (coded as "1"), "no" (coded as "0"), or "not sure" (coded as "0.5") to capture uncertainty. Following case presentation, subtypes and risk factors were determined by majority vote, with all responses collected anonymously via the online platform.

**Longitudinal classification and subtype assignment:**

For participants with multiple consensus assessments (baseline, middle, and/or late follow-up), ARF subtype was determined through a two-step process:

***Step 1 - Timepoint-specific classification:*** At each assessment (baseline, middle, late), participants were independently classified into one of the seven ARF subtypes based on clinical data, imaging, and physiology available at that specific timepoint. These timepoint-specific classifications were recorded for each participant.

***Step 2 - Overall hospitalization assignment:*** The final ARF subtype for the hospitalization was determined using a hierarchical approach that prioritized ARDS identification. If a participant met criteria for ARDS at any timepoint (baseline, middle, or late), they were assigned to the ARDS subtype for all study analyses presented in this manuscript, regardless of their classification at earlier timepoints. Similarly, participants classified as ARFA at one or more timepoints were only assigned to the ARFA subtype overall if they never progressed to meet ARDS criteria during any follow-up assessment. This hierarchical assignment ensures that ARDS and ARFA represent mutually exclusive "destination" subtypes for this analysis. In our cohort, 1% (n=2) of patients with an overall ARDS assignment were initially classified as ARFA at baseline but progressed to meet ARDS criteria at a subsequent timepoint. Conversely, to receive an overall ARFA assignment, patients must have maintained risk factors for acute lung injury without ever fulfilling ARDS diagnostic criteria throughout the entire observation period. This classification strategy reflects our intent to capture the most severe ARF phenotype experienced during the acute illness, which we hypothesized would have the greatest relevance for long-term outcomes.

**Biomarker measurements and assessment of biologic subphenotypes:**

Participants underwent collection of blood biospecimens upon enrollment for centrifugation and separation of plasma (stored at -80^o^C). Host-response biomarkers shown to have validated associations with ARDS and/or sepsis had been previously determined from plasma samples using a customized 10-plex Luminex assay (R&D Systems, Minneapolis).[^2^](https://sciwheel.com/work/citation?ids=7657922&pre=&suf=&sa=0&dbf=0) We assigned host-response subphenotypes (hyperinflammatory vs. hypoinflammatory) using a previously validated parsimonious logistic regression model developed and externally validated by our group. This four-biomarker model was originally derived using latent class analysis (LCA) followed by feature selection with a best subsets generalized linear model (using 100-fold cross-validation) in a subset of 498 mechanically ventilated patients from the present cohort who were enrolled between 2011-2020 and had complete biomarker data.[^4^](https://sciwheel.com/work/citation?ids=12208454&pre=&suf=&sa=0&dbf=0) The parsimonious model incorporates baseline plasma procalcitonin (ng/mL), angiopoietin-2 (Ang-2, pg/mL), soluble tumor necrosis factor receptor-1 (sTNFR1, pg/mL), and serum bicarbonate (CO₂, mEq/L). The logistic regression equation is:

**logit(P) = -2.367566 - 0.2379745 × (bicarbonate) + 0.0006844 × (procalcitonin) + 0.0004073 × (sTNFR1) + 0.00010378 × (angiopoietin-2)**

where P represents the predicted probability of hyperinflammatory phenotype. Biomarker concentrations and bicarbonate values are entered as raw, untransformed values in their respective units. The predicted probability was calculated as P = e^logit(P) / (1 + e^logit(P)) and dichotomized at a threshold of 50%: participants with P ≥ 50% were classified as hyperinflammatory, and those with P < 50% as hypoinflammatory. Importantly, we did not perform re-calibration or modify model covariates. The model was applied using the original published coefficients to evaluate its validity and prognostic performance in the expanded ARF cohort.

**Missing data handling:**

Missing data were handled using clinically informed imputation strategies that leveraged established physiologic relationships and clinical knowledge Biomarker values were never imputed (see main Methods). Below we detail imputation methods for ventilatory parameters, blood gas measurements, and clinical laboratory values.

Respiratory Mechanics

Missing ventilatory parameters were imputed using the following hierarchy:

1. Plateau Pressure (24% missingness): Missing plateau pressure values were imputed using multiple imputation with predictive mean matching (30 imputations). The imputation model incorporated physiologically related ventilatory parameters (peak inspiratory pressure, PEEP, tidal volume, respiratory rate, FiO2, PaO2/FiO2 ratio, ventilator mode) and patient characteristics (age, sex, BMI). Predictive mean matching was selected to ensure imputed values were drawn from the observed data distribution, maintaining biological plausibility. Physiologic constraints (PEEP < plateau pressure < peak pressure) were enforced for all imputed values.
2. Peak Inspiratory Pressure (4% missingness): When PIP was missing but PEEP was available (indicating the patient was mechanically ventilated), PIP was imputed as 26.5 cm H₂O (cohort mean).
3. PEEP (0.3% missingness: When PEEP was missing but PIP was available, PEEP was imputed as 8 cm H₂O (cohort mean).

After imputation and calculations, driving pressure remained unavailable for 34 (4.1%) participants, representing incomplete ventilator data documentation.

Gas Exchange Parameters

1. PaO₂/FiO₂ Ratio (35.3% missingness): When arterial blood gas PaO₂ was unavailable, we estimated PaO₂/FiO₂ from SpO₂/FiO₂ using a validated non-linear conversion model previously developed and published by our group.[^12^](https://sciwheel.com/work/citation?ids=13131766&pre=&suf=&sa=0&dbf=0) This model accounts for the non-linear relationship between oxygen saturation and partial pressure across the physiologic range.
2. PaCO₂ (38.9% missingness): When arterial PaCO₂ was missing but venous blood gas PvCO₂ was available, we imputed: PaCO₂ = PvCO₂ - 4 mmHg, based on the established physiologic difference between venous and arterial CO₂ tension in critically ill patients.[^13^](https://sciwheel.com/work/citation?ids=4793484&pre=&suf=&sa=0&dbf=0) This imputed an additional 113 (13.7%) missing values. Ventilatory ratio was estimated in these complete cases post-imputation.

Clinical Laboratory Values

Missingness for routine laboratory values was minimal (<1%). We used within-patient imputation for participants with multiple study timepoints, imputing missing baseline values using the mean of available measurements from the same patient:

- Creatinine: 4 missing at baseline (0.5%), all 4 successfully imputed (0 remained missing)
- Hemoglobin: 2 missing at baseline (0.2%), 1 imputed, 1 remained missing (0.1%)
- Platelets: 4 missing at baseline (0.5%), 3 imputed, 1 remained missing (0.1%)
- White blood cells: 4 missing at baseline (0.5%), 3 imputed, 1 remained missing (0.1%)
- Bicarbonate: 3 missing at baseline (0.4%), 2 imputed, 1 remained missing (0.1%)

After within-patient imputation, residual missingness was negligible (0-1 case per variable, <0.2% each). Complete-case analysis was used for these few participants with persistently missing values.

**Expanded Results for Inter-Rater Reliability Assessments:**

Assessment of inter-rater reliability:

We analyzed data for 331 subjects reviewed in survey-based consensus meetings, which totaled 70 hourly meetings (average of 4.7 subjects reviewed per meeting). Ten different reviewers participated, with a minimum of 3 reviewers per meeting (median 4, IQR 3-7). A sample de-identified case with standardized data presentation is provided in Supplementary file 1. We examined inter-rater agreement for: a) radiographic assessment, b) ARF subtype classification, and c) risk factors for lung injury (e-Table 3).

a) Radiographic assessments: Overall, inter-rater agreement was high, with an intraclass correlation co-efficient -ICC of 0.92 (95% confidence interval 0.73-0.99). By majority vote, 67.3% of radiographs (CXRs and/or CTs) were deemed consistent with ARDS, while 32.7% were not. Unanimous agreement was more common for radiographs consistent with ARDS (i.e. all votes “yes”, 87.4%) compared to those not consistent with ARDS (i.e. all votes “no”, 63.9%, Fisher p<0.001). Unanimous agreement for ARDS radiographic presentations was also higher among patients with COVID-19 (83.3%) compared to non-COVID ARF patients (37.5%, p<0.001).
b) ARF subtype classifications: The 331 subjects were classified as 46.2% ARDS, 37.6% ARFA, 2.9% CHF, 4.1% Airway Controls, 0.1% AoCHRF, 2.4% AEILD, and 5.9% Other/Multifactorial. We found that classifications into Airway Control, AEILD and ARDS subtypes had the highest agreement rates (92.9%, 87.5% and 84.5% unanimous agreements among reviewers), with lower proportions of unanimous agreements for other subtypes, especially CHF and Other (e-Figure 2).
c) Risk factor assessments: Among the 331 subjects analyzed, 294 (88.8%) were deemed to have ≥1 risk factor for lung injury by at least one reviewer, with 66 (19.9%) and 12 (3.6%) were considered to have either 2 or 3 risk factors, respectively. Pneumonia was the most assigned risk factor (72.9% of all risk factor assignments), followed by extra-pulmonary sepsis (12.6%) and aspiration (7.3%) (e-Figure 3).

**Supplemental Tables and Figures**

**e-Table 1: Inclusion and exclusion criteria for the Acute Lung Injury Registry and Biospecimen Repository**

**Inclusion Criteria**

1. Adult patients between 18 and 90 years of age inclusive.
2. Admission to the intensive care unit with acute respiratory failure with one or more the following:

a. A risk factor for developing acute lung injury including pneumonia, sepsis, gastric aspiration, pancreatitis, blood transfusion, traumatic injury,
 or major surgery.
 b. Airway protection for non-respiratory causes including toxic overdoses, seizure, gastrointestinal bleeding, post-surgical purposes, or
 cerebrovascular accidents.
 c. Cardiogenic pulmonary edema.

d. Exacerbation of chronic obstructive lung disease.
 e. Interstitial lung disease.

**Exclusion criteria**

1. Inability to provide informed consent either directly or through their proxy.
2. Acute or chronic respiratory failure due to neuromuscular disease.
3. Chronic respiratory failure as defined by tracheotomy or chronic ventilator dependence.

**e-Table 2: Operational definitions of direct and indirect risk factors for lung injury adopted from original publications that resulted in inclusion into the Berlin definition.**

| **Direct Risk Factors** | **Definition** |
| --- | --- |
| 1. Pneumonia | Clinical diagnosis of pneumonia, as supported airspace opacities on chest imaging, purulent sputum, abnormal temperature (< 36 or > 38°C), or white blood cell count (WBC > 12,000, < 4,000 or > 10% bands). We also performed implicit review of the EMR physicians’ documentation regarding their bedside consideration of pneumonia diagnosis at time of presentation. We did not mandate positive microbiology for pneumonia diagnosis but recorded cases that had respiratory and/or blood cultures or other microbiologic testing. |
| 2. Aspiration | Macro-Aspiration, which was supported by a witnessed or documented aspiration event or the retrieval of gastric contents from the oropharynx, endotracheal tube, or bronchial tree, as deduced from review of presenting illness history, and intubation or bronchoscopy procedural documentation. We did not consider the mentioning of “aspiration” in clinical/radiology notes as diagnostic of macro-aspiration, as the term is frequently used in clinical practice based on suspicion of aspiration without objective evidence.[^6^](https://sciwheel.com/work/citation?ids=14818913&pre=&suf=&sa=0&dbf=0) Importantly, we distinguished Macro-Aspiration as a risk factor for lung injury from the subsequent development of pneumonia, which occurs in a subset of patients who experience macro-aspiration. Patients who developed pneumonia following a macro-aspiration event would be assigned risk factors for both aspiration and pneumonia, whereas patients who experienced macro-aspiration without subsequent evidence of pneumonia would be only assigned the aspiration risk factor. |
| 3. Inhalational injury | Any type of inhalational insult known to be associated with lung injury, including vaping, illicit drug use, or fire smoke inhalation. |
| 4. Pulmonary contusion | Present on imaging as distinct pulmonary opacities in patients with trauma or post prolonged cardiopulmonary resuscitation efforts. |
| 5. Pulmonary Vasculitis | Diagnosis of pulmonary vasculitis by positive autoantibodies or biopsy. |
| 6. Drowning | Salt or freshwater aspiration with drowning. |
| **Indirect Risk Factors** | **Definition** |
| 1. Non-pulmonary sepsis | Sepsis diagnosis (clinical diagnosis of infection with end-organ damage) that is not from pulmonary origin. |
| 2. Major trauma | Blunt or open abdominal/chest trauma, or large bone fractures. |
| 3. Pancreatitis | History consistent with acute pancreatitis, elevated lipase levels and consistent imaging findings on CT abdomen. |
| 4. Severe burns | Partial or full thickness burns of ≥ 20% total body surface area. |
| 5. Non-cardiogenic shock | Hypotension and tissue hypoperfusion from mechanism other than cardiogenic shock, e.g., severe vasoplegic shock from acute liver failure. For cases with septic shock, we assigned the non-pulmonary sepsis or pneumonia (direct risk factor) as indicated, instead of the “non-cardiogenic shock” variable. |
| 6. Drug overdose | Overdose by opiates, cocaine, or amphetamines. |
| 7. Multiple transfusions or transfusion-associated  acute lung injury (TRALI) | Massive transfusion: Transfusion of ≥ 4 units of packed red blood cells within 24hrs.  TRALI: transfusion of fresh frozen plasma with resultant acute lung injury and without another identifiable risk factor for lung injury. |

**e-Figure 1: Schematic of the online survey-based consensus process:** A study investigator conducts a standardized case presentation in Microsoft PowerPoint slides to a panel of physician-scientist reviewers via a secure Microsoft Teams meeting. The reviewers have access to a standardized online questionnaire with de-identified data (Qualtrics) and are asked to answer questions during the virtual meeting. Each case presentation begins with the radiographic criterion question (Q1, i.e. *is this radiographic presentation consistent with ARDS?)* before any clinical data are provided. Reviewers are first shown the admitting chest x-ray (CXR), the CXR on day of enrollment, and the oldest CXR available in the electronic medical record (EMR) for comparison. If a thoracic computed tomography (CT) was obtained within 72hrs from enrollment, then selected cuts at 3 levels were also demonstrated. Reviewers then answer Q1 as “yes”, “no” or “not sure”. Following Q1, a detailed presentation of clinical data follows, with review of narrative information from admitting and progress notes by house staff and attending physicians at time of enrollment, gas exchange data, vital signs, clinical microbiology, and cardiovascular system assessments (echocardiography, right heart catheterization data, fluid balance, BNP levels, as available). Reviewers are then given the opportunity to ask additional clarifying questions and then answer Q2 question acute respiratory failure subtype classification (*What is the appropriate acute respiratory failure subtype for this subject?)* and Q3 regarding the identified risk factors for lung injury (*Does this subject have risk factors for lung injury?).* Acute respiratory failure subtype and risk factors are determined by majority of votes for each participant.

**e-Table 3: Survey-based consensus sample questionnaire:**

| **Question** | **Possible Answers** |
| --- | --- |
| Does Sample Day 1 CXR (Case X) **have bilateral opacities consistent with ARDS** (i.e., pulmonary edema not fully explained by effusions, lobar or lung atelectasis, or nodules or masses)? | 1. Yes 2. No 3. Not Sure 4. No CXR available |
| What is your best classification of this subtype of Acute Respiratory Failure on Day 1? | 1. ARDS 2. At-risk for ARDS 3. CHF 4. Airway control 5. Acute on chronic hypercapnic respiratory failure 6. Acute on exacerbation of ILD 7. Other 8. cannot classify in any of the above categories- I need more info |
| Does this patient have risk factors for ARDS (check all that apply)? | Direct   1. Pneumonia 2. Aspiration 3. Inhalational injury 4. Pulmonary contusion 5. Pulmonary Vasculitis 6. Drowning   Indirect   1. Non-pulmonary sepsis 2. Major trauma 3. Pancreatitis 4. Severe burns 5. Non-cardiogenic shock 6. Drug overdose 7. TRALI |

**e-Figure 2: Unanimous agreement for ARF subtype classifications was variable among different ARF subtypes.** All subjects were classified by majority vote to one of the following seven subtypes. AEILD, Airway Controls and ARDS had the highest proportions of unanimous agreement in their classifications (i.e. all reviewers voted the same ARF subtype).

**e-Figure 3: Total number of risk factor assignments by individual reviewers.**

**e-Figure 4: Temporal Trends in Patient Enrollment and ARF Subtype Distribution Across Study Epochs.**

**A. Density plot showing the distribution of patient enrollment over the study period (2011-2024), with vertical dashed lines demarcating three study epochs: First Epoch (2011-2016), Second Epoch (2017-2019), and Third Epoch (2020-2024). B. Proportional stacked bar chart showing the distribution of all seven ARF subtypes across the three study epochs. Numbers within each segment indicate the count of patients in that subtype. The prevalence of ARDS (red) increased markedly in the third epoch, coinciding with the COVID-19 pandemic. C. ARDS prevalence by study epoch, demonstrating significant temporal variation: 37% (91/248 patients) in the first epoch, 20% (65/323 patients) in the second epoch, and 55% (142/259 patients) in the third epoch (χ² test for trend, p<0.001). Pairwise comparisons between epochs were all statistically significant (p<0.001). While formal enrollment criteria remained consistent, the surge in ARDS prevalence during 2020-2024 primarily reflects the COVID-19 pandemic's impact on ICU case mix, whereas variation in earlier epochs reflects both evolving enrollment priorities and changes in ARF population characteristics.**

**e-Figure 5.** **Ventilator management by ARF subtype.** Day 1 tidal volumes (A) and PEEP (B) differed significantly across subtypes. AoCHRF patients received higher tidal volumes (median 7.4 mL/kg of ideal body weight), whereas PEEP was highest in ARDS and AEILD (10.0 and 8.0 cm H₂O, respectively). Boxes show IQRs with median lines; points represent individual patients; p-values from pairwise Wilcoxon tests for comparisons with ARDS with asterisks denoting statistical significance (p<0.05)

**e-Figure 6. Use of advanced organ support therapies by ARF subtype.**
Stacked barplots show the proportion of patients within each acute respiratory failure (ARF) subtype receiving (A) extracorporeal membrane oxygenation (ECMO), (B) continuous neuromuscular blockade (NMBA), and (C) continuous renal replacement therapy (CRRT) during the ICU stay. ECMO and NMBA use were concentrated among patients with ARDS and AEILD, whereas CRRT was most common in ARDS. Percentages represent the proportion of patients receiving each therapy within subtype.

**e-Figure 7. Gas exchange and respiratory mechanics differences between ARF subtypes.** Analyses exclude patients on ECMO.

**e-Figure 8. Radiographic characteristics across acute respiratory failure subtypes.**
Boxplots show RALE scores from the first post-intubation chest radiograph (top).

**e-Figure 9. Echocardiographic findings by acute respiratory failure (ARF) subtype.**
Boxplots show (A) left atrial diameter, (B) left ventricular diastolic diameter, and (C) left ventricular ejection fraction (LVEF) measured during the ICU stay among 427 participants with available echocardiograms. CHF patients demonstrated significantly larger left atrial and diastolic diameters and lower LVEF compared with ARDS (all p < 0.05). No other subtype comparisons reached statistical significance. Data are displayed as individual points with overlaid boxplots; p-values are based on pairwise comparisons between each subtype and ARDS. Proportion of patients with available echocardiographic measurements by ARF subtype: ARDS 57%, ARFA 47%, CHF 77%, Airway Controls 27%, AoCHRF 44%, AEILD 58%, other 64%.

**e-Figure 10: Plasma biomarker differences across subtypes.**

**e-Table 4. Baseline Characteristics of Participants With Complete vs. Incomplete Biomarker Data**

| Characteristic | Complete Biomarker Data (n=745) | Incomplete Biomarker Data (n=81) |
| --- | --- | --- |
| Demographics |  |  |
| Age, years, median [IQR] | 58.7 [46.8, 67.9] | 60.2 [49.0, 67.9] |
| Male sex, n (%) | 424 (56.9) | 50 (61.7) |
| BMI, kg/m², median [IQR] | 29.7 [25.6, 36.0] | 29.3 [24.2, 35.6] |
| Severity of Illness |  |  |
| SOFA score, median [IQR] | 7.0 [5.0, 9.0] | 7.0 [4.0, 11.0] |
| Vasopressor requirement, n (%) | 592 (79.5) | 55 (67.9) |
| Comorbidities |  |  |
| Diabetes mellitus, n (%) | 260 (34.9) | 23 (28.4) |
| COPD, n (%) | 149 (20.0) | 20 (24.7) |
| Chronic heart failure, n (%) | 97 (13.0) | 12 (14.8) |
| Immunosuppression, n (%) | 149 (20.0) | 25 (30.9) |
| ARF Subtype, n (%) |  |  |
| ARDS | 265 (35.6) | 33 (40.7) |
| At-Risk for ARDS | 257 (34.5) | 25 (30.9) |
| CHF | 48 (6.4) | 3 (3.7) |
| Airway Control | 78 (10.5) | 10 (12.3) |
| AoCHRF | 27 (3.6) | 0 (0.0) |
| AEILD | 18 (2.4) | 1 (1.2) |
| Other/Multifactorial | 52 (7.0) | 9 (11.1) |
| Outcomes |  |  |
| 3-year survival, n (%) | 388 (52.1) | 41 (50.6) |
| Duration of mechanical ventilation, days, median [IQR] | 6.0 [3.0, 13.0] | 6.0 [3.0, 11.2] |

ARDS = acute respiratory distress syndrome; ARFA = at-risk for ARDS; CHF = congestive heart failure; AoCHRF = acute-on-chronic hypercapnic respiratory failure; AEILD = acute exacerbation of interstitial lung disease; COPD = chronic obstructive pulmonary disease; SOFA = Sequential Organ Failure Assessment; IQR = interquartile range.

**e-Table 5: Plasma biomarkers by ARF subtype, excluding patients on ECMO (expressed in pg/ml of plasma).**

| Biomarker | AoCHRF (n=27) | AEILD (n=17) | Airway Control (n=88) | ARDS (n=268) | ARFA (n=279) | CHF (n=51) | Other (n=59) |
| --- | --- | --- | --- | --- | --- | --- | --- |
| Interleukin-6 | 11.8 [6.2, 35.5] | 24.9 [9.4, 114] | 35.6 [19.1, 69.4] | 116 [29.8, 356] | 66.1 [26.4, 201] | 54.9 [27.0, 118] | 35.8 [19.1, 72.3] |
| Interleukin-8 | 12.3 [6.0, 21.7] | 18.0 [8.6, 25.7] | 8.7 [5.4, 20.0] | 23.8 [14.2, 43.1] | 20.3 [9.4, 41.2] | 13.1 [7.8, 22.4] | 13.4 [7.1, 24.5] |
| Interleukin-10 | 2.0 [0.8, 7.8] | 1.0 [0.3, 1.7] | 1.3 [0.4, 2.2] | 1.8 [1.0, 9.3] | 2.2 [0.8, 11.5] | 1.3 [0.8, 2.3] | 1.6 [1.1, 5.6] |
| sTNFR1 | 2,376 [1,662, 3,215] | 2,367 [1,786, 3,147] | 2,152 [1,356, 3,997] | 4,840 [2,942, 8,820] | 4,579 [2,633, 8,520] | 5,602 [3,126, 10,258] | 4,328 [2,598, 8,304] |
| sST2 | 158,662 [118,647, 373,646] | 164,890 [80,188, 422,719] | 71,869 [39,019, 241,857] | 165,987 [84,517, 371,370] | 251,051 [96,043, 775,081] | 151,339 [95,692, 350,240] | 141,460 [65,212, 292,169] |
| Fractalkine | 1,633 [1,026, 2,722] | 1,894 [1,006, 2,646] | 766 [159, 1,514] | 2,187 [1,210, 3,624] | 1,784 [735, 2,857] | 1,585 [855, 2,556] | 1,660 [628, 2,869] |
| Pentraxin-3 | 2,914 [1,192, 8,960] | 2,402 [1,786, 4,300] | 1,468 [845, 4,199] | 7,393 [3,509, 18,827] | 6,461 [2,185, 18,216] | 3,480 [1,154, 10,401] | 4,202 [1,799, 12,971] |
| Procalcitonin | 195 [73.7, 638] | 354 [145, 710] | 159 [70.3, 581] | 798 [333, 3,053] | 1,185 [304, 4,041] | 1,038 [385, 2,792] | 603 [183, 1,773] |
| sRAGE | 1,573 [1,008, 2,707] | 1,445 [537, 2,591] | 1,777 [1,261, 2,719] | 5,467 [2,977, 9,927] | 3,629 [2,211, 6,626] | 4,643 [3,322, 7,899] | 3,256 [2,130, 5,838] |
| Angiopoietin-2 | 3,451 [2,242, 8,261] | 4,385 [2,697, 6,993] | 3,489 [2,072, 5,871] | 7,901 [3,914, 15,375] | 8,679 [4,197, 17,811] | 7,592 [4,663, 15,463] | 6,474 [4,259, 12,302] |

**e-Figure 11: Duration of invasive mechanical ventilation differed significantly among subtypes, with ARDS patients requiring the longest median ventilation time (10 days).**

**e-Figure 12: Discharge destinations for survivors of the acute hospitalization.** Destinations are displayed hierarchically according to functional independence, from home (most independent) to long-term acute care hospitals and skilled nursing facilities (least independent). Airway control and AoCHRF subtypes were most frequently discharged to independent settings (>50%), whereas ARDS patients most often required prolonged post-acute care.

**Figure 13. Three-Year Survival by Study Epoch.** Three-year survival rates remained stable across study epochs (2011-2016, 2017-2019, 2020-2024; p for trend = 0.09) with numerically lower survival in the latest epoch during the COVID-19 pandemic. Numbers indicate percentage and counts (survivors/total).

**e-Figure 14. Schoenfeld Residual Plots for Assessment of Proportional Hazards Assumption.** **Scaled Schoenfeld residual plots for ARF subtype in (A) unadjusted and (B) adjusted Cox models for three-year survival. The solid line represents a smoothed estimate of the time-varying coefficient; dashed lines show ±2 standard errors. Residuals are randomly distributed around zero without systematic temporal patterns. The proportional hazards assumption was satisfied (p=0.25 and p=0.23, respectively), supporting the validity of Cox proportional hazards modeling for survival analyses.**

**e-Figure 15: Association Between Acute Life Support Interventions and Short-Term vs. Long-Term Survival.** Kaplan-Meier survival curves stratified by receipt of neuromuscular blockade (NMBA), continuous renal replacement therapy (CRRT), or vasopressors during ICU stay. Left panels show 90-day survival from ICU admission for patients receiving NMBA (top), CRRT (middle), or vasopressors (bottom). All three interventions were strongly associated with early mortality (log-rank p≤0.002 for each), reflecting their use as markers of disease severity. Right panels show long-term survival among hospital survivors through 3 years post-discharge. Among patients who survived to hospital discharge, receipt of these intensive therapies during acute illness was not associated with differences in long-term survival (log-rank p>0.05 for each), suggesting that initial severity requiring these interventions does not portend worse outcomes among those who survive the acute hospitalization. Number at risk displayed below each curve.

**Supplemental File 1**

**Sample Case Presentation**

**Slide 1**

Disclaimer

- Patient data presentation is confidential and for purposes of disease phenotyping
- This file should be kept behind the OneDrive firewall at all times

**Slide 2**

XXX ID XXXX

Name: XXXXXX

Medical Record Number: XXXXXX

Date of ICU admission: XXXXXX

Date of intubation: XXXXXX

Sample 1 Date: XXXXXX

Sample 5 Date: XXXXXX

Sample 10 Date: XXXXXX

**Slide 3**

CXR at intubation

**Slide 4**

CXR at Day 1 Sample

**Slide 5**

Oldest CXR available

*No prior CXRs available*

**Slide 6**

Consensus Question 1

Does Sample Day 1 CXR (XXXX XXXX) **have bilateral opacities consistent with ARDS** (i.e. pulmonary edema not fully explained by effusions, lobar or lung atelectasis, or nodules or masses?)

**Slide 7**

Gas exchange data

|  | XXXXXX | XXXXXX | XXXXXX |
| --- | --- | --- | --- |
| pHa | 7.38 | 7.48 | 7.4 |
| PaCO2 | 47 | 36 | 46 |
| PaO2 | 64 | 41 | 72 |
| HCO3 | 27 | 26 | 28 |

Sample Day 1 P/F ratio: 64/0.6 = 107

**Slide 8**

*HPI and Impression from Resident Note on Sample Day 1 - v*

XXXXXX *with PMH of HTN, kidney cysts, and prostate cancer who presents with acute hypoxemic respiratory failure 2/2 COVID PNA with progression to ARDS prompting transfer to MICU* XXXXXX *for worsening hypoxemia, now paralyzed and proned.*

*#Acute hypoxemic respiratory failure 2/2 COVID ARDS*

*- Positive test on* XXXXXX *s/p remdesivir x 3 doses, toci, vanc/cefepime* XXXXXX *Intubated redacted, now with severe ARDS requiring paralysis and proning. Recruitable so weaning O2 on higher PEEP.*

*- P:F 120 on* XXXXXX *prompting proning. Repeat VBG w/ P:F 200, will wean PEEP to 10, plan to supine this afternoon and re-assess oxygenation*

*- continuing low tidal volume ventilation and permissive hypercapnia in favor of lung protection*

*- 10 day course dexamethasone*

*- fluid balance: goal net even today. If able to wean off NE, will aim for net neg tomorrow*

*#Leukocytosis*

*#Hypotension*

*-WBC trending up to 22k, persistent NE requirements. Likely i/s/o inflammatory response 2/2 COVID19*

*- BCx* XXXXXX *NGTD, sputum Cx* XXXXXX *NGTD*

*- BAL cultures* XXXXXX *pending*

*- finished course of cefepime* XXXXXX

*#Htn: holding losartan and Aldactone*

*#BPH: continue tamsulosin*

***Slide 9***

*Attending Impression on Sample 1 Date –* XXXXXX

*The key issues:*

*> Acute respiratory failure – ARDS secondary to COVID-19. Cannot rule out complicating bacterial superinfection. Severe gas exchange requirement requiring paralysis and prone positioning. Poor compliance in prone position though oxygenation improved. Targe low volume ventilation and allow permissive hypercapnia in favor of lung protection. Oxygenation markedly improved but required bronchoscopy for mucus plug extraction overnight. Add hypertonic saline nebs. Maintain supine position when turned later today.*

*> COVID-19 pneumonia: Daily dexamethasone. Received tocilizumab and remdesivir. Prophylactic doses of enoxaparin.* XXXXXX*. Bacterial cultures pending, already received 5 days of broad spectrum antibiotics.*

*> Hypotension: In context of systemic inflammatory response syndrome and sedation. Wean Norepi as able.*

**Slide 10**

SIRS Criteria

**Slide 11**

Microbiologic cultures for specimens obtained in the 72hr period from intubation

Respiratory:

- ETA: r XXXXXX
  - Gram stain: many WBCs present; few epithelial cells; few gram + cocci in pairs and in clusters; few gram variable rods; few yeast
  - Culture: Many yeast, heavy normal flora
- BAL XXXXXX
  - Gram stain: few WBCs, no organisms present
  - Cultures: no growth x 2 days
- Blood cultures: XXXXXX
  - No growth in two days
- Respiratory viral panel: not performed
- Other micro data: VRE/MRSA not isolated

**Slide 12**

Cardiovascular Data

TTE not done

BNP not done

**Slide 13**

Consensus Question 2:

Case XXXXXX Consensus

Day 1 classification of acute respiratory failure:

1. ARDS
2. At-risk for ARDS
3. CHF
4. Airway control
5. Acute on chronic hypercapneic respiratory failure
6. Acute on chronic ILD
7. Other
8. I cannot classify in any of the above categories – I need more information

**Slide 14**

Consensus Question 3:

Did this patient have risk factors for ARDS?

Direct

1. Pneumonia
2. Aspiration
3. Inhalational injury
4. Pulmonary contusion
5. Pulmonary vasculitis
6. Drowning

Indirect

a. Non-pulmonary sepsis
b. Major trauma
c. Pancreatitis
d. Severe burns
e. Non-cardiogenic shock
f. Drug overdose
g. TRALI

**Slide 15**

Consensus Question 4

What is your best guess about this patient’s subphenotypic classification on Day 1?

1. Hyperinflammatory
2. Hypoinflammatory
3. Not sure

**Slide 16**

Day 5 CXR – XXXXXX

**Slide 17**

Consensus Question 5

Does Sample Day 5 CXR (XXXXXX) **have bilateral opacities consistent with ARDS** (i.e. pulmonary edema not fully explained by effusions, lobar or lung atelectasis, or nodules or masses?)

**Slide 18**

HPI and Impression from Resident Note on Sample Day 5- XXXXXX

XXXXXX with PMH of htn, kidney cysts, and prostate cancer who presents with acute hypoxemic respiratory failure 2/2 COVID PNA with progression to ARDS prompting transfer to MICU XXXXXX for worsening hypoxemia, s/p paralytic and proning, now improving.

#Acute hypoxemic respiratory failure 2/2 COVID ARDS

- Positive test on XXXXXX, s/p remdesivir x 3 doses, toci, vanc/cefepime (XXXXXX). Intubated XXXXXX, now with severe ARDS requiring paralysis and proning. Recruitable so weaning O2 on higher PEEP.

- Currently on VC, FiO2 60%, PEEP 10

- off paralytics since XXXXXX, off versed, now on fentanyl and propofol for sedation

- attempt to wean FiO2 to 50% as able, keep PEEP at 10 since PEEP responsive, SBT once FiO2 improved

- continuing low tidal ventilation and permissive hypercapnia in favor of lung protection

- s/p 10 day course of dexamethasone

- s/p 7d course cefepime

- fluid balance: goal net even, wean off of norepinephrine XXXXXX, Cx remain negative so far. Poor windows on POCUS, unable to assess volume status/cardiac fxn but appears to be euvolemic

- HD lovenox

- Repeating CXR and sputum culture for evaluation of superimposed infection

Chronic medical issues:

#Htn: holding losartan and Aldactone

#BPH: continue tamsulosin

**Slide 19**

Attending Impression on Sample 5 Date

Acute respiratory failure requiring mechanical ventilation

COVID ARDS

Severe asynchrony with HIGH air hunger

Weaning FiO2 down to 60%, TV 6 mL/kg IBW

Low dose NE infusion to keep MAP > 65

**Slide 20**

Additional data

Sputum cx redacted Few WBCs, no organisms seen

Cx rare NRF

**Slide 21**

Consensus Question 6

Case XXXXXX Consensus

Day 5 classification of acute respiratory failure:

1. ARDS
2. At-risk for ARDS
3. CHF
4. Airway control
5. Acute on chronic hypercapneic respiratory failure
6. Acute on chronic ILD
7. Other
8. I cannot classify in any of the above categories – I need more information

**Slide 22**

Consensus Question 7

Did this patient have new risk factors for ARDS on Day 5?

Direct

1. Pneumonia
2. Aspiration
3. Inhalational injury
4. Pulmonary contusion
5. Pulmonary vasculitis
6. Drowning

Indirect

a. Non-pulmonary sepsis
b. Major trauma
c. Pancreatitis
d. Severe burns
e. Non-cardiogenic shock
f. Drug overdose
g. TRALI

**Slide 23**

Consensus Question 8

What is your best guess about this patient’s subphenotypic classification on Day 5?

1. Hyperinflammatory
2. Hypoinflammatory
3. Not sure

**Slide 24**

Day 10 CXR – XXXXXX

**Slide 25**

Consensus Question 9

Does Sample Day 10 CXR (XXXXXX) **have bilateral opacities consistent with ARDS** (i.e. pulmonary edema not fully explained by effusions, lobar or lung atelectasis, or nodules or masses?)

**Slide 26**

HPI and Impression from Resident Note on Sample Day 10

XXXXXX PMH of htn, kidney cysts, prostate Ca here w/ ARDS 2/2 COVID pna.

#AHRF 2/2 COVID ARDS, intubated XXXXXX

COVID+ XXXXXX, s/p remdesivir x 3, toci, vanc/cefepime XXXXXX, dex x10d. Intubated XXXXXX, c/b severe ARDS requiring paralysis and proning. s/p cefepime x 7d. Appears volume up on POCUS. Consider new pna given new fever, leukocytosis, incr pressor req.

- Nimbex gtt for paralysis, fentanyl/midaz/ketamine gtt for sedation

- s/p proning XXXXXX, supinated XXXXXX, plan to prone again today

#Shock

Likely septic given new fever, leukocytosis. Less likely cardiogenic although tn 0.2, EKG with evolving ST changes; bedside POCUS w/ good EF, no wall motion abn, e/o tamponade/RV strain. DDx includes AI, anaphylaxis appears less likely. Appears volume overloaded on POCUS; will defer IVF for now.

- Repeat EKG pending, tn downtrended peak 0.33

- Levo gtt MAP > 65

- Vanc/cefepime restarted, BCx, SCx, CXR pending

**Slide 27**

**Attending Impression on Sample 10 Date**

XXXXXX with prostate Ca and severe COVID PNA/ARDS

- worsening gas exchange and work of breathing

- Remains supine and paralyzed, will resume proning

- resumed broad spectrum antibiotics given leukocytosis, fever, and worsening infiltrates

**Slide 28**

Consensus Question 10

Case XXXXXX Consensus

Day 10 classification of acute respiratory failure:

1. ARDS
2. At-risk for ARDS
3. CHF
4. Airway control
5. Acute on chronic hypercapneic respiratory failure
6. Acute on chronic ILD
7. Other
8. I cannot classify in any of the above categories – I need more information

**Slide 29**

Consensus Question 11

Did this patient have new risk factors for ARDS on Day 10?

Direct

7. Pneumonia
8. Aspiration
9. Inhalational injury
10. Pulmonary contusion
11. Pulmonary vasculitis
12. Drowning

Indirect

a. Non-pulmonary sepsis
b. Major trauma
c. Pancreatitis
d. Severe burns
e. Non-cardiogenic shock
f. Drug overdose
g. TRALI

**Slide 30**

Consensus Question 12

What is your best guess about this patient’s subphenotypic classification on Day 10?

1. Hyperinflammatory
2. Hypoinflammatory
3. Not sure

References:

[1.    Labar AS, Ulrich BC, Lovelace TC, et al. Plasma levels of soluble ST2 reflect extrapulmonary organ dysfunction and predict outcomes in acute respiratory failure. Crit Care Med 2025;53(8):e1567–e1580.](https://sciwheel.com/work/bibliography/17902524)

[2.    Kitsios GD, Yang L, Manatakis DV, et al. Host-Response Subphenotypes Offer Prognostic Enrichment in Patients With or at Risk for Acute Respiratory Distress Syndrome. Crit Care Med 2019;47(12):1724–1734.](https://sciwheel.com/work/bibliography/7657922)

[3.    Bain W, Yang H, Shah FA, et al. COVID-19 versus Non-COVID-19 Acute Respiratory Distress Syndrome: Comparison of Demographics, Physiologic Parameters, Inflammatory Biomarkers, and Clinical Outcomes. Ann Am Thorac Soc 2021;18(7):1202–1210.](https://sciwheel.com/work/bibliography/10433421)

[4.    Drohan CM, Nouraie SM, Bain W, et al. Biomarker-Based Classification of Patients With Acute Respiratory Failure Into Inflammatory Subphenotypes: A Single-Center Exploratory Study. Crit Care Explor 2021;3(8):e0518.](https://sciwheel.com/work/bibliography/12208454)

[5.    Lu M, Drohan C, Bain W, et al. Trajectories of Host-Response Subphenotypes in Patients With COVID-19 Across the Spectrum of Respiratory Support. CHEST Critical Care 2023;1(3).](https://sciwheel.com/work/bibliography/15886182)

[6.    Kitsios GD, Nguyen VD, Sayed K, et al. The upper and lower respiratory tract microbiome in severe aspiration pneumonia. iScience 2023;26(6):106832.](https://sciwheel.com/work/bibliography/14818913)

[7.    Kitsios GD, Sayed K, Fitch A, et al. Longitudinal multicompartment characterization of host-microbiota interactions in patients with acute respiratory failure. Nat Commun 2024;15(1):4708.](https://sciwheel.com/work/bibliography/16531108)

[8.    ARDS Definition Task Force, Ranieri VM, Rubenfeld GD, et al. Acute respiratory distress syndrome: the Berlin Definition. JAMA 2012;307(23):2526–2533.](https://sciwheel.com/work/bibliography/585910)

[9.    Heijnen NFL, Hagens LA, Smit MR, et al. Biological Subphenotypes of Acute Respiratory Distress Syndrome Show Prognostic Enrichment in Mechanically Ventilated Patients without Acute Respiratory Distress Syndrome. Am J Respir Crit Care Med 2021;203(12):1503–1511.](https://sciwheel.com/work/bibliography/12208446)

[10.   Collard HR, Ryerson CJ, Corte TJ, et al. Acute exacerbation of idiopathic pulmonary fibrosis. an international working group report. Am J Respir Crit Care Med 2016;194(3):265–275.](https://sciwheel.com/work/bibliography/1837087)

[11.   Ferguson ND, Fan E, Camporota L, et al. The Berlin definition of ARDS: an expanded rationale, justification, and supplementary material. Intensive Care Med 2012;38(10):1573–1582.](https://sciwheel.com/work/bibliography/1761451)

[12.   Ren S, Zupetic JA, Tabary M, et al. Machine learning based algorithms to impute PaO2 from SpO2 values and development of an online calculator. Sci Rep 2022;12(1):8235.](https://sciwheel.com/work/bibliography/13131766)

[13.   Nassar BS, Schmidt GA. Estimating arterial partial pressure of carbon dioxide in ventilated patients: how valid are surrogate measures? Ann Am Thorac Soc 2017;14(6):1005–1014.](https://sciwheel.com/work/bibliography/4793484)
