# Supplementary material for: Characterization and 3-Year Survival of Clinical Subtypes and Host Response Phenotypes of Acute Respiratory Failure Requiring Mechanical Ventilation
Source: CHEST Crit Care. Author manuscript; Available in PMC 2026 Oct 1. (PMC13626120; doi:10.1016/j.chstcc.2026.100274)
Supplement: Supplementary file 2 [file NIHMS2210626-supplement-2.docx]

STROBE Statement Checklist

Observational Cohort Study

| Item No. | Recommendation | Page No. |
| --- | --- | --- |
| 1(a) | Indicate the study's design with a commonly used term in the title or the abstract | 3 |
| 1(b) | Provide in the abstract an informative and balanced summary of what was done and what was found | 3-4 |
|  | **INTRODUCTION** |  |
| 2 | Explain the scientific background and rationale for the investigation being reported | 5 |
| 3 | State specific objectives, including any prespecified hypotheses | 5 |
|  | **METHODS** |  |
| 4 | Present key elements of study design early in the paper | 6 |
| 5 | Describe the setting, locations, and relevant dates, including periods of recruitment, exposure, follow-up, and data collection | 6, Supplement |
| 6(a) | Cohort study—Give the eligibility criteria, and the sources and methods of selection of participants. Describe methods of follow-up | 6, Supplement |
| 6(b) | Cohort study—For matched studies, give matching criteria and number of exposed and unexposed | N/A |
| 7 | Clearly define all outcomes, exposures, predictors, potential confounders, and effect modifiers. Give diagnostic criteria, if applicable | 6-8 |
| 8 | For each variable of interest, give sources of data and details of methods of assessment (measurement). Describe comparability of assessment methods if there is more than one group | 6-8, Supplement |
| 9 | Describe any efforts to address potential sources of bias | 8-9, 16 |
| 10 | Explain how the study size was arrived at | 9 |
| 11 | Explain how quantitative variables were handled in the analyses. If applicable, describe which groupings were chosen and why | 8-9, Supplement |
| 12(a) | Describe all statistical methods, including those used to control for confounding | 8-9 |
| 12(b) | Describe any methods used to examine subgroups and interactions | 9 |
| 12(c) | Explain how missing data were addressed | 8, Supplement |
| 12(d) | Cohort study—If applicable, explain how loss to follow-up was addressed | 9, 15 |
| 12(e) | Describe any sensitivity analyses | Supplement |
|  | **RESULTS** |  |
| 13(a) | Report numbers of individuals at each stage of study—eg numbers potentially eligible, examined for eligibility, confirmed eligible, included in the study, completing follow-up, and analysed | 9-10, Supplement |
| 13(b) | Give reasons for non-participation at each stage | Supplement |
| 13(c) | Consider use of a flow diagram | N/A |
| 14(a) | Give characteristics of study participants (eg demographic, clinical, social) and information on exposures and potential confounders | 10-11, Table 2 |
| 14(b) | Indicate number of participants with missing data for each variable of interest | Supplement |
| 14(c) | Cohort study—Summarise follow-up time (eg, average and total amount) | 11 |
| 15 | Cohort study—Report numbers of outcome events or summary measures over time | 11-12, Table 3 |
| 16(a) | Give unadjusted estimates and, if applicable, confounder-adjusted estimates and their precision (eg, 95% confidence interval). Make clear which confounders were adjusted for and why they were included | 11-12, Table 3 |
| 16(b) | Report category boundaries when continuous variables were categorized | Supplement |
| 16(c) | If relevant, consider translating estimates of relative risk into absolute risk for a meaningful time period | 11-12 |
| 17 | Report other analyses done—eg analyses of subgroups and interactions, and sensitivity analyses | 12, Figure 3 |
|  | **DISCUSSION** |  |
| 18 | Summarise key results with reference to study objectives | 12-13 |
| 19 | Discuss limitations of the study, taking into account sources of potential bias or imprecision. Discuss both direction and magnitude of any potential bias | 15-16 |
| 20 | Give a cautious overall interpretation of results considering objectives, limitations, multiplicity of analyses, results from similar studies, and other relevant evidence | 12-16 |
| 21 | Discuss the generalisability (external validity) of the study results | 15-16 |
|  | **OTHER INFORMATION** |  |
| 22 | Give the source of funding and the role of the funders for the present study and, if applicable, for the original study on which the present article is based | Title page |
